# Advanced malignancies treated with a combination of the VEGF inhibitor bevacizumab, anti-EGFR antibody cetuximab, and the mTOR inhibitor temsirolimus

**DOI:** 10.18632/oncotarget.7594

**Published:** 2016-02-24

**Authors:** Xiaochun Liu, Susan Kambrick, Siqing Fu, Aung Naing, Vivek Subbiah, George R. Blumenschein, Bonnie S. Glisson, Merrill S. Kies, Apostolia M. Tsimberidou, Jennifer J. Wheler, Ralph G. Zinner, David S. Hong, Razelle Kurzrock, Sarina A. Piha-Paul

**Affiliations:** ^1^ Department of Investigational Cancer Therapeutics, University of Texas, MD Anderson Cancer Center, Houston, Texas, USA; ^2^ Department of Thoracic/Head & Neck Medical Oncology, University of Texas, MD Anderson Cancer Center, Houston, TX, USA; ^3^ Division of Hematology and Oncology, University of California San Diego, Moores Cancer Center, San Diego, CA, USA

**Keywords:** solid tumors, bevacizumab, cetuximab, temsirolimus

## Abstract

**BACKGROUND:**

Bevacizumab and temsirolimus are active agents in advanced solid tumors. Temsirolimus inhibits mTOR in the PI3 kinase/AKT/mTOR pathway as well as CYP2A, which may be a resistance mechanism for cetuximab. In addition, temsirolimus attenuates upregulation of HIF-1α levels, which may be a resistance mechanism for bevacizumab.

**RESULTS:**

The median age of patients was 60 years (range, 23-80 years). The median number of prior systemic therapies was 3 (range, 1-6). The maximum tolerated dose (MTD) was determined to be bevacizumab 10 mg/kg biweekly, temsirolimus 5 mg weekly and cetuximab 100/75 mg/m2 weekly. Grade 3 or 4 toxicities were seen in 52% of patients with the highest prevalence being hyperglycemia (14%) and hypophosphatemia (14%). Eighteen of the 21 patients were evaluable for response. Three patients were taken off the study before restaging for toxicities. Partial response (PR) was observed in 2/18 patients (11%) and stable disease (SD) lasting ≥ 6 months was observed in 4/18 patients (22%) (total = 6/18 (33%)). In 8 evaluable patients with squamous cell carcinoma of the head and neck (HNSCC) there were partial responses in 2/8 (25%) patients and SD ≥ 6 months in 1/8 (13%) patients (total = 3/8, (38%)).

**PATIENTS AND METHODS:**

We analyzed safety and responses in 21 patients with advanced solid tumors treated with bevacizumab, cetuximab, and temsirolimus.

**CONCLUSION:**

The combination of bevacizumab, cetuximab, and temsirolimus showed activity in HNSCC; however, there were numerous toxicities reported, which will require careful management for future clinical development.

## INTRODUCTION

Epidermal growth factor receptor (EGFR), vascular endothelial growth factor (VEGF), and mammalian target of rapamycin (mTOR) are important targets in a wide array of malignancies [1]. Cetuximab is a monoclonal antibody that inhibits EGFR signaling, resulting in inhibition of cell growth, induction of apoptosis, and decreased matrix metalloproteinase and VEGF production [2]. It has been approved by the US Food and Drug Administration (FDA) to treat colorectal cancer as well as head and neck cancer [3]. Bevacizumab is a monoclonal antibody specific for the VEGF family of proteins and receptors that are important in tumor angiogenesis and fundamental for tumor growth and metastasis [4–8]. Bevacizumab has been approved by the FDA to treat metastatic cancers including colorectal cancer, non-small cell lung cancer, glioblastoma, cervical cancer, and renal cell carcinoma [9]. Temsirolimus is an mTOR inhibitor that inhibits the phosphoinositide 3-kinase (PI3K)/Protein kinase B (AKT)/mTOR pathway, which is involved in protein synthesis, cellular proliferation, and tumor angiogenesis [10, 11]. Temsirolimus has been approved by the FDA to treat renal cell carcinoma [12].

A given tumor is unlikely to be dependent upon only one receptor or signaling pathway for its growth and survival. This is due to the significant level of compensatory cross talk among receptors within a signaling network as well as heterologous receptor systems [13, 14]. Therefore, combining drugs inhibiting different signaling pathways is currently an important strategy to achieve synergy or overcome resistance.

The synergy between the VEGF and EGFR pathways lies in their close relationship and sharing common downstream signaling pathways [15]. Activation of EGFR signaling in tumor cells stimulates the production of VEGF, which then acts in a paracrine fashion on surrounding endothelial cells to stimulate their proliferation and migration [16, 17]. Combinations of VEGF(R) and EGFR inhibitors have shown synergy in antitumor activities in lung cancer and colorectal cancer in preclinical studies [18–20]. In a phase II clinical study of 46 patients with squamous cell carcinoma of the head and neck (HNSCC), bevacizumab in combination with cetuximab achieved an objective response rate of 16% and a disease control rate of 73% [21, 22]. The median progression-free survival and overall survival were 2.8 and 7.5 months, respectively.

One mechanism of tumor resistance to antiangiogenic therapy (e.g. bevacizumab) is upregulation of hypoxia-inducible factor 1α (HIF-1α), which mediates adaptive responses to hypoxic conditions [6, 23–30]. HIF-1α inhibition in combination with antiangiogenic therapy is a promising strategy for targeting tumor resistance [27, 31–34]. Temsirolimus has been shown to inhibit the activity of mTOR and has resulted in reduced levels of HIF-1α, HIF-2α and VEGF [30]. The discovery of the HIF-1α inhibition properties of temsirolimus makes it an ideal candidate for combination with bevacizumab. In a phase I clinical study of 41 heavily-pretreated patients with gynecological malignancies, 37% achieved disease control [21].

It has been suggested that downstream PI3K-AKT-mTOR hyperactivation may represent an acquired mechanism of resistance to EGFR inhibition that can be overcome by combination therapy. In addition, temsirolimus enhances anti-cancer efficacy of cetuximab through the inhibition of protein phosphatase 2A(CYP2A) in preclinical models of colorectal cancer [35]. Temsirolimus added to cetuximab in cetuximab-refractory head and neck cancer patients induced responses in 16.7% of subjects [36]. Therefore, adding temsirolimus to the combination may abrogate resistance to both bevacizumab and cetuximab.

Taken together, there are several compelling rationales for combining bevacizumab, temsirolimus, and cetuximab in treating advanced malignancies: i) bevacizumab and cetuximab may be synergistic; ii) temsirolimus inhibits mTOR and the PI3 kinase/AKT/mTOR pathway as well as CYP2A, which may be a resistance mechanism for cetuximab; iii) temsirolimus attenuates upregulation of HIF-1α levels, which may be a resistance mechanism for bevacizumab; and iv) the three agents have non-overlapping toxicities. Here we report our experience treating patients with advanced malignancies with this combination therapy.

## RESULTS

### Demographic and clinical characteristics

Twenty-one patients with advanced, metastatic malignancies were enrolled between March 2012 and February 2014. Demographic and clinical characteristics are summarized in Table 1. The median age of patients was 60 years (range, 23-80 years). The median number of prior systemic therapies was 3 (range, 1-6). Before enrollment onto the trial, three patients had received prior mTOR inhibitors (2 patients had received temsirolimus and 1 patient had received everolimus), four patients had received prior bevacizumab, and seven patients had received prior cetuximab. The most common cancer type was HNSCC followed by melanoma. All patients had experienced disease progression on their prior therapy. The median number of cycles (cycle = 28 days) completed for all patients was 2 (range, 1-10). Ten patients (48%) received more than 2 cycles. For patients with SD or better, the median number of cycles completed was 4 (range, 1-10).

**Table 1:** Baseline demographics and clinical characteristics (N=21)

Patient Characteristic	Total (%)
Age, years	
Median (Range)	60 (23-80)
Gender	
Male	13 (62%)
Female	8 (38%)
ECOG performance status^*^	
0	4 (19%)
1	17 (81%)
Number of prior systemic therapies	
Median (Range)	3 (1-6)
Number of prior temsirolimus	2 (10%)
Number of prior bevacizumab	4 (19%)
Number of prior cetuximab	7 (33%)
Tumor type	
Colorectal	1
Squamous cell carcinoma head & neck	9 (43%)
Melanoma	2 (10%)
Chondrosarcoma	1
Cholangiocarcinoma	1
Mesothelioma	1
Cervical adenoma	1
Adenocarcinoma of tongue	1
Adenocarcinoma of Gastroesophageal Junction	1
Gastric adenocarcinoma	1
Adnexal carcinoma	1
Urethral carcinoma	1

* ECOG = Eastern Cooperative Oncology Group

### Toxicity assessment

Patient enrollment proceeded in accordance with the planned 3+3 study design (Table 2). Three patients were enrolled in dose level 1. The toxicities seen in dose level 1 included grade 3 neutropenia along with grade 1-2 anemia, dermatitis, epistaxis, fatigue, hypercholesterolemia, hyperglycemia, hyperkalemia, hypertriglyceridemia, hypokalema, hypophosphatemia, elevated AST/ALT, renal insufficiency, mucositis/glossitis, nausea, vomiting, proteinuria, fistula, hypomagnesemia and pneumonitis (Table 3). No DLTs were observed in dose level 1. Three patients were then enrolled in dose level 2. No DLTs were obtained in dose level 2. At dose level 3, our first patient experienced two DLTs (grade 3 mucositis and grade 3 fatigue) and the second patient enrolled experienced a DLT (grade 3 headache) as well. Therefore, the enrollment for dose level 3 was discontinued per protocol. We enrolled 3 additional patients to confirm that dose level 2 was safe and after verifying tolerability, enrolled 10 additional patients to further evaluate toxicity and preliminary anti-tumor efficacy as an MTD expansion cohort. The toxicities based on a total of 16 patients treated at dose level 2 were: grade 4 stomach leak/perforation; grade 3 hypophosphatemia, elevated AST, hyperglycemia, hypo- or hyperkalemia, anemia, infusion reaction and vomiting; and, grade 1-2 anemia, dermatitis, epistaxis, fatigue, hypercholesterolemia, hyperglycemia, hyperkalemia, hypertriglyceridemia, hypokalema, hypophosphatemia, elevated AST/ALT, renal insufficiency, infection, neutropenia/leukopenia, mucositis/glossitis, nail discoloration, nausea, proteinuria, dyspnea with exertion, stomatitis, thrombocytopenia, abdominal pain, anorexia, diarrhea, dysuria, fever, headache, hypertension and hyperbilirubinemia (Table 3). Among these, grade 4 stomach leak/perforation, grade 3 elevated AST, and grade 3 infusion related reaction are considered DLTs. However, it is important to note that these 3 DLTs occurring in 3 separate patients did not meet the criteria for MTD as the total number of DLTs occurred in less than 33% of patients (3/16 patients or 19%). Per protocol, dose level 2 (bevacizumab 10 mg/kg biweekly, temsirolimus 5 mg weekly and cetuximab 100/75 mg/m2 weekly) was defined as the maximum tolerated dose (MTD).

**Table 2:** Dose-Escalation Schedule (28-day cycle), Grade 3/4 Toxicities^*^ and Response

Dose Level	N	Temsirolimus IV on Days 1, 8, 15, 22	Bevacizumab IV on Days 1, 15	Cetuximab IV on Days 1, 8, 15, 22	SD≥6 months or PR /Total treated	Grade (G) 3/4 Toxicity (N)^*^
1	3	5 mg	5 mg/kg	100/75 mg/m^2^	1/3	G3/4 Neutropenia (1)
2	16	5 mg	10 mg/kg	100/75 mg/m^2^	5/16	G3 Hypophosphatemia (1) G3 Elevated Aspartate Aminotransferase (1)^Δ^ G3 Hyperglycemia (3) G3 Hypokalemia (1) G3 Anemia (1) G3 Hyperkalemia (1) G3 Infusion reaction (1)^Δ^ G3 Vomiting (1) G4 Stomach leak/perforation (1)^Δ^
3	2	12.5 mg	2.5 mg/kg	100/75 mg/m^2^	0/2	G3 Headache (1)^Δ^ G3 Hypophosphatemia (2) G3 Mucositis (1)^Δ^ G3 Fatigue (1)^Δ^

* Adverse events deemed at least possibly related to treatment were graded based on the Common Terminology Criteria for Adverse Events, version 3.0 (CTCAEv3.0)

Δ was defined as a dose-limiting toxicity

Abbreviations: N, number of patients

**Table 3:** Adverse events at any dose level

Adverse Event of All Grades	Dose Level of Temsirolimus (mg)/Bevacizumab (mg/kg)/Cetuximab LD/MD (mg/m^2^)							
	5/5/100/75 (n = 3)		5/10/100/75 (n = 16)		12.5/2.5/100/75 (n = 2)		Total (n = 21)	
	G1-2	G3-4	G1-2	G3-4	G1-2	G3-4	G1-2	G3-4
Abdominal Pain	0	0	1	0	0	0	1	0
Anemia	3	0	13	1	2	0	18	1
Anorexia	0	0	2	0	2	0	4	0
Dermatitis	1	0	10	0	1	0	12	0
Diarrhea	0	0	1	0	0	0	1	0
Dysuria	0	0	1	0	0	0	1	0
Epistaxis	1	0	2	0	0	0	3	0
Fatigue	2	0	8	0	1	1	11	1
Fever	0	0	1	0	0	0	1	0
Headache	0	0	4	0	1	1	5	1
Hypercholesterolemia	2	0	9	0	0	0	11	0
Hyperglycemia	3	0	8	3	1	0	12	3
Hyperkalemia	1	0	2	1	2	0	5	1
Hypertension	0	0	2	0	0	0	2	0
Hypertriglyceridemia	3	0	11	0	1	0	15	0
Hypokalemia	1	0	4	1	1	0	6	1
Hypophosphatemia	1	0	3	1	0	2	4	3
Elevated AST	3	0	8	1	0	0	11	1
Hyperbilirubinemia	0	0	1	0	0	0	1	0
Renal Insufficiency	1	0	3	0	0	0	4	0
Elevated ALT	2	0	6	0	1	0	9	0
Infection (ear, orbit)	1	0	3	0	0	0	4	0
Leukopenia/Neutropenia	0	1	5	0	1	0	6	1
Mucositis/Glossitis	2	0	8	0	0	1	10	1
Nail Discoloration	0	0	2	0	0	0	2	0
Nausea	1	0	3	0	2	0	6	0
Proteinuria	2	0	11	0	0	0	13	0
Dyspnea with Exertion	0	0	1	0	0	0	1	0
Stomatitis	0	0	2	0	0	0	2	0
Thrombocytopenia	0	0	3	0	2	0	5	0
Fistula	1	0	0	0	0	0	1	0
Hypomagnesemia	1	0	0	0	0	0	1	0
Pneumonitis	1	0	0	0	0	0	1	0
Vomiting	1	0	0	1	2	0	3	1
Oral thrush	0	0	0	0	1	0	1	0
Constipation	0	0	0	0	1	0	1	0
Infusion Reaction	0	0	0	1	0	0	0	1
Stomach leak/rupture	0	0	0	1	0	0	0	1

LD: loading dose; MD: maintenance dose.

All 21 patients with advanced malignancy experienced at least one adverse event that was possibly related to the drug. These events were mostly grade 1 or 2 and reversible. In fact, 10 patients (48%) experienced toxicity no greater than grade 2. Grade 3 or 4 toxicities were as follows: hyperglycemia (14%), hypophosphatemia (14%), headache (5%), hyperkalemia (5%), hypokalemia (5%), fatigue (5%), elevated aspartate aminotransferase (AST) (5%), decreased absolute neutrophil count/leucopenia (5%), mucositis (5%), anemia (5%), infusion reaction (5%), vomiting (5%), and stomach leak/perforation (5%) (Table 3). Among the toxicities, six DLTs were observed in five patients at two dose levels: grade 3 elevated AST, grade 3 infusion reaction, and grade 4 stomach leak/rupture (patient had gastric antral mass) in three separate patients at dose level 2 (bevacizumab 10 mg/kg and temsirolimus 5 mg, cetuximab 100/75 mg/m^2^ weekly); grade 3 fatigue and grade 3 mucositis in one patient and grade 3 headache (not posterior reversible encephalopathy syndrome (PRES)) in another patient at dose level 3 (bevacizumab 2.5 mg/kg and temsirolimus 12.5 mg, cetuximab 100/75 mg/m^2^ weekly) (Table 2). Three patients (14%) came off study before the first restaging due to toxicities: grade 4 stomach leak/rupture, grade 3 headache, and grade 3 infusion reaction, respectively. There were no thromboembolic events or cases of significant proteinuria. Of the 16 patients treated at the MTD, one patient (6%) was dose-reduced for toxicities occurring during the first cycle. In this case, the cetuximab was dose reduced by 50% because of grade 3 elevated AST.

### Antitumor activity

**Figure 1:**
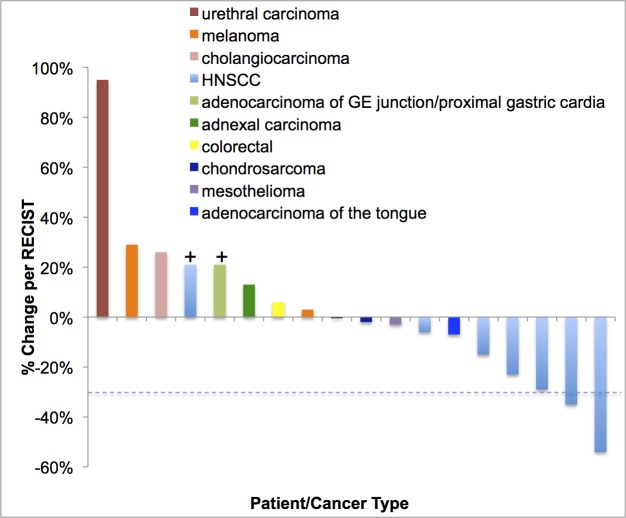
Waterfall plot depicting best RECIST response by patient Individual patients/disease sites are represented by vertical bars on the X-axis. The best RECIST response (%) is depicted on the Y-axis. Sixteen of the 21 patients were measurable by RECIST. Two patients were assigned a value of +21% for clinical progression or new lesions (+). Three patients are not included in this plot as they never reached restaging due to being taken off the study early in the first cycle for toxicity. Dotted line shows 30% response by RECIST.

Of 21 total patients on the trial, 16/21 (76%) patients had disease that was measurable by RECIST and reached restaging; 2/21 (10%) patients had clinical progression; and 3/21 (14%) patients were taken off the study before restaging for toxicities. For the purposes of reporting, 18/21 (86%) patients were considered evaluable for response. Figure 1 is a waterfall plot depicting best response of the 18 patients. Partial response (PR) was observed in 2/18 patients (11%); and stable disease (SD) lasting ≥ 6 months was observed in 4/18 patients (22%) (total = 6/18 (33%) with SD ≥ 6 months/PR). Details regarding these patients including dose level, duration of treatment, and best response by RECIST 1.0 are described in Table 4.

**Table 4:** Stable disease ≥ 6 months or partial response (PR) by RECIST and characterization by patient

	Cancer Type	Dose Level	Best Response by RECIST 1.0	# of Prior Cytotoxic Regimens	Duration of Treatment (Weeks)	*PTEN* mut^*^	*PIK3CA* mut	*RAS* mut	*RAF* mut	*P53* mut
9^ζ^	HNSCC	1	−29%	2	40	N	N	N	N	N
35	Chondrosarcoma	2	−2%	4	40	N	N	N	N	N
42^Δ^	Adenocarcinoma of the tongue	2	−7%	5	32	N	N	N	N	Y
46^ζ^	HNSCC	2	−54%	2	16	N	N	N	N	N
48	HNSCC	2	−35%	3	28	N	N	N	N	N
59	Melanoma	2	+3%	3	32	N	N	N	N	ND

Abbreviations: N, no mutation; Mut, mutation; ND, not done; HNSCC, squamous cell carcinoma of the head and neck; P, present.

* PTEN loss by immunohistochemistry was not performed.

Δ indicates a patient with prior temsirolimus and bevacizumab treatment.

ζ indicates a patient with prior cetuximab treatment.

**Figure 2:**
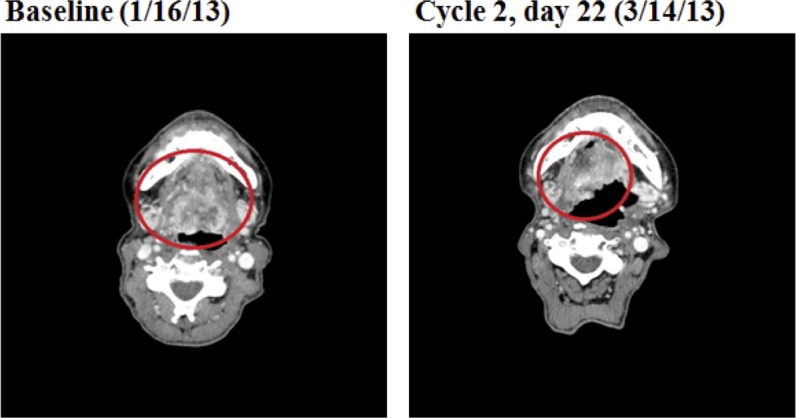
A patient with HNSCC showed partial response (−54%) on the first restaging scan The baseline and the first restaging CT scan of a 60-year-old woman with squamous cell carcinoma of the base of the tongue who had received prior cetuximab.

Of 8 patients with evaluable HNSCC, SD ≥ 6 months/PR was achieved in 3 (38%) patients. These included two PRs (one patient with cancer arising from the base of the tongue with prior exposure to cetuximab (Figure 2) and one patient with cancer arising from the left tonsil with no prior exposure to cetuximab) and one SD for 10 months (in a patient with cancer arising from the oral tongue with prior exposure to cetuximab). Human papilloma virus (HPV) test was negative in the patient with HNSCC from the base of the tongue who achieved PR for 4 months. HPV testing was equivocal in the patient with HNSCC from the left tonsil who achieved PR for 7 months. Finally, for the HNSCC achieving SD for 10 months whose disease arose from the oral tongue, HPV testing was negative. The 1/1 patient with adenocarcinoma of the tongue attained prolonged SD for 8 months. It was also observed that 1/2 melanoma patients and 1/1 chondrosarcoma patient achieved prolonged SD for 8 months and 10 months, respectively.

### Molecular analysis and association with response

When archival cell blocks for patients were available, CLIA-certified testing was performed for *BRAF, NRAS, KRAS, HRAS, PTEN, P53* and *PIK3CA* mutations along with evaluation for PTEN loss by IHC. Fourteen out of 21 patients (67%) had known mutational status (Table 5). *PIK3CA* mutational status was positive in 1/14 (7%) patients and this patient had progressive disease. *PTEN* mutation was found in 2/14 patients (14%) tested; both patients were off study before restaging due to toxicities. Only one patient (7%) had IHC testing and confirmed PTEN loss; this patient had 23% decrease of target lesions as the best response and eventually progressed after 3 cycles of treatment. Of all 14 patients with *RAS* and *RAF* mutational analysis, only one patient (7%) was positive for *HRAS* mutation. This patient had progressive disease. *P53* is a predictor of response to bevacizumab [37]. In addition, Schwaederle et al. demonstrated that *P53* mutations correlate strongly with increased VEGF-A, the target of bevacizumab [38]. In our study, we had two patients with *P53* aberration. The first patient had adenocarcinoma of gastroesophageal junction/proximal gastric cardia, which was positive for *TP53* Y103*, a deletion/insertion in exon 4 of the *TP53* gene, which is predicted to result in a stop codon with early termination of translation. This patient had progressive disease at cycle 1 day 21. The second patient had adenocarcinoma of the tongue that was positive for *TP53* R213*, which is a hotspot mutation. This patient had stable disease for 8 cycles.

**Table 5:** Tumor molecular analysis

Tumor Molecular Aberration	N/Total tested (%)	Cancer Type	Best Response Comments
*PIK3CA* (C378F)	1/14 (7%)	Cholangiocarcinoma	26% increase
*PTEN* (R173C)	1/14 (7%)	Cervical cancer	NE; off study for grade 3 headache after first bevacizumab infusion
*PTEN* (R335*)	1/14 (7%)	HNSCC	NE; off study for grade 3 infusion reaction during first cetuximab infusion
*PTEN* loss	1/14 (7%)	HNSCC	23% decrease
*HRAS* (Q61R)	1/14 (7%)	Urethral carcinoma	95% increase

Abbreviation: N, number of patients; NE, no response evaluation; HNSCC, squamous cell carcinoma of the head and neck; IHC, immunohistochemistry

## DISCUSSION

This is the first study to evaluate the combination of bevacizumab, temsirolimus, and cetuximab in patients with advanced malignancies. This combination demonstrated promising activity, but at the expense of toxicity. Overall, 11/21 (52%) of patients treated on the trial developed grade 3 to 4 toxicities including: hyperglycemia (14%), hypophosphatemia (14%), headache (5%), hyperkalemia (5%), hypokalemia (5%), fatigue (5%), elevated aspartate aminotransferase (5%), decreased absolute neutrophil count/leukopenia (5%), mucositis (5%), anemia (5%), infusion reaction (5%), vomiting (5%), and stomach leak/perforation (5%). A total of five patients (24%) experienced DLTs. Three of these patients (14%) discontinued treatment before the first restaging due to DLTs (one patient with HNSCC; one patient with gastric cancer; and one patient with cervical adenocarcinoma). The most frequent grade 3 to 4 toxicities (observed in ≥ 10% of patients) were hyperglycemia and hypophosphatemia.

The most common non-hematologic adverse events (observed in ≥ 50% of patients) were dermatitis, fatigue, hypercholesterolemia, hyperglycemia, hypertriglyceridemia, elevated AST, mucositis, and proteinuria. Hyperglycemia and hyperlipidemia have been reported as common adverse events after temsirolimus treatment, affecting 17-26% and 6-27% treated patients respectively [39, 40]. Seventy-one percent of our patients developed hyperglycemia and hypertriglyceridemia. Dermatitis occurs in 47-75% of patients treated with temsirolimus [39–41] and in 78% of patients treated with cetuximab [42]. We observed dermatitis in 57% of our patients, which is actually lower than the reported incidence for either single agent temsirolimus or cetuximab. Fatigue occurs in 71% of patients treated with temsirolimus [43] and in 63% of patients treated with cetuximab [44]. Fatigue was observed in 57% of our patients, which was lower than the reported incidence for single agent temsirolimus, but higher than that seen for single agent cetuximab. Mucositis was observed in 52% of our patients, which is slightly higher than the 46% incidence rate reported in a phase 3 trial of single agent temsirolimus in renal cell carcinoma.[40] Proteinuria has been reported to occur in 32% of ovarian cancer patients treated with single agent bevacizumab. [45] Proteinuria was observed at a higher rate in our patients at 62%.

The most common hematologic toxicity was anemia, which occurred in 19 (90%) patients. We also observed neutropenia/leukopenia and thrombocytopenia in 7 (33%) and 5 (24%) treated patients, respectively. Grade 3/4 neutropenia and thrombocytopenia are rare with temsirolimus, cetuximab, or bevacizumab. In our treated patients, we observed only one patient with anemia and no patients with thrombocytopenia. None of our patients developed thromboembolic complications. In the current study, bevacizumab (10 mg/kg IV once every 14 days), temsirolimus (5 mg IV weekly), and cetuximab 100/75 mg/m^2^ IV weekly) was determined to be the MTD. With the exception of bevacizumab, the other two drugs were dosed well below their label indication including, temsirolimus at 20% and cetuximab at 40% of the FDA approved doses. This reflects synergistic toxicity that could limit further development of this combination.

Evidence of clinical benefit in patients with HNSCC was observed. One of 9 HNSCC patients discontinued therapy after the first cetuximab infusion due to grade 3 infusion reaction. Of the remaining eight evaluable patients, seven patients made it to restaging and one patient was taken off therapy after cycle 1 due to clinical progression. This yielded an overall response rate of 25% (2/8) and a disease control rate of 38% (3/8). The median progression free survival (PFS) was 3 months in all eight evaluable patients with HNSCC and 1.8 months in all 21 patients on the trial. Anti-cancer activity has been observed with the combination of bevacizumab and cetuximab in HNSCC in both preclinical xenograft models and in a phase II study [22]. This phase II study yielded a comparable objective response rate of 16% but a higher disease control rate of 73%. The median progression-free survival was 2.8 months; however, the patient population of that study was targeting locally advanced squamous cell carcinoma with only one line of prior therapy. This study also excluded patients treated with either prior cetuximab or bevacizumab. The toxicity profile in this phase II study was better compared to our study likely due to a less frequent dosing schedule. In addition, prolonged SD ≥ 6months was also observed in one of each patient with chondrosarcoma, melanoma, and adenocarcinoma of the tongue.

There are other clinical trials published in the literature that have combined three targeted agents [46–48]. For example, in a phase 1 trial, 34 patients with non-small cell lung cancer (NSCLC) received the combination of cetuximab, erlotinib and bevacizumab and were included in a subgroup analysis. [47] Unlike our trial, this combination was overall well-tolerated in these NSCLC patients. Of the NSCLC patients in this trial, the most common treatment-related grade ≥ 2 adverse events were rash (14/34, 41%), hypomagnesemia (9/34, 27%), and fatigue (5/34, 15%). The antitumor activity in these NSCLC patients, however, is similar to our study with seven patients (21%) achieving stable disease (SD) ≥ 6 months, two patients (6%) achieving a partial response (PR) and two patients (6%) achieving an unconfirmed partial response (uPR) (total = 11/34, (32%)) in heavily pretreated patients. In another phase 1 trial, 32 patients with different types of solid tumors received the combination of everolimus, bevacizumab and panitumumab. [48] This trial was also overall well tolerated and appeared to have moderate clinical activity in refractory tumors. Common adverse events were skin rash/pruritus (29/32, 91 %), mucositis/stomatitis (24/32, 75 %), hypomagnesemia (23/32, 72 %), hypocalcemia (18/32, 56 %) and hypokalemia (16/32, 50 %). There were 3 partial responses; an additional 10 subjects had stable disease ≥ 6 months. Three subjects with ovarian cancer and one with endometrial cancer achieved prolonged disease control ranging from 11 to > 40 months.

Our study has several limitations. Firstly, a biomarker was not elucidated because molecular analysis could not be performed in many patients due to lack of tissue for testing. Secondly, the response signals may be limited because these patients were heavily pre-treated, with a median of three prior systemic therapies. Lastly, we were limited in total number of patients without prior exposure to single agent cetuximab, bevacizumab, or temsirolimus. The correlation between prior response to single agent and the efficacy of combination therapy could not be established.

In conclusion, the combination of bevacizumab, temsirolimus, and cetuximab showed activity in HNSCC; however, numerous toxicities were reported, which would require careful management during future clinical development.

## PATIENTS AND METHODS

### Study design and dosing

The experience with advanced malignancies reported was a single institution, phase I, open-label, dose-escalation study. This trial was open to all patients with advanced or metastatic cancer refractory to standard therapy, relapsed after standard therapy, or who had no standard therapy available that could improve survival by at least three months.

Treatment was administered on an outpatient basis at the University of Texas, MD Anderson Cancer Center (UTMDACC). A cycle of therapy was 28 days. No investigational, commercial agents or therapies other than those described here could be administered with the intent to treat the patient's malignancy. Bevacizumab was given on days 1 and 15 of each cycle, while temsirolimus and cetuximab was given weekly on days 1, 8, 15, and 22 (Table 2). Restaging scans were performed after every two cycles. Consent was obtained and patients were treated in accordance with UTMDACC Institutional Review Board guidelines.

The protocol followed a standard 3+3 design. If one patient in a cohort experienced a dose-limiting toxicity (DLT) during the first cycle, three additional patients were enrolled and treated at that dose level. If at any time more than 33% of patients in a cohort experienced a DLT, that cohort was closed to additional patients. Adverse events were graded based on the Common Terminology Criteria for Adverse Events, version 3.0 (CTCAEv3.0). DLTs were defined as any grade three or four non-hematologic toxicity that was believed to be related to any of the study medications (except nausea and vomiting responsive to appropriate regimens, correctable electrolyte imbalances or alopecia); any Grade 4 hematologic toxicity lasting 2 weeks or longer (as defined by the CTCAEv3.0) despite supportive care; any Grade 4 nausea or vomiting > 5 days despite maximum anti-nausea regimens; any other Grade 3 non-hematologic toxicity including symptoms/signs of vascular leak or cytokine release syndrome; any severe or life-threatening complication/abnormality not defined in the CTCAEv3.0 that was attributable to the therapy. The MTD was defined by DLTs that occurred in the first cycle (four weeks). The use of growth factors was accepted during the clinical study.

### Eligibility criteria

Key inclusion criteria were histologically-documented, advanced or metastatic solid tumors refractory to standard treatment or for which no standard therapy was available; Eastern Cooperative Oncology Group (ECOG) performance status ≤ two; absolute neutrophil count ≥ 1 × 10^9^/L; platelet count ≥ 50.0 × 10^9^/L; serum creatinine < 3.0 mg/dL, aspartate transferase (AST), alanine transferase (ALT) ≤ five times the upper limit of normal (ULN); bilirubin ≤ 3.0 mg/dL, total fasting cholesterol ≤ 350 mg/dL; and triglyceride ≤ 400 mg/dL. Key exclusion criteria were clinically significant, unexplained bleeding or hemoptysis within 28 days prior to study entry; poorly controlled hypertension (systolic blood pressure > 140 mm Hg, diastolic pressure > 90 mm Hg on medication); patients with clinically significant cardiovascular disease; *KRAS* mutated colorectal cancer patients; history of hypersensitivity to any of the three drugs; patients who had major surgery within 6 weeks of enrollment in the study; and pregnancy. Patients with prior exposure to bevacizumab, cetuximab or mTOR inhibitors were not excluded from the study, nor were patients with a history of venous thromboembolism excluded.

### Assessment of tumor response

Tumor measurements were performed on patients with measurable disease at baseline and every two cycles thereafter. Measurable target lesions were evaluated for response using Response Evaluation Criteria in Solid Tumors (RECIST 1.0) [49, 50]. For the purpose of this report, prolonged stable disease (SD) was defined as lasting ≥ 6 months.

### Molecular analysis (*PIK3CA, PTEN, RAF* and *RAS*)

*PIK3CA, RAF, RAS* mutations were investigated in archival formalin-fixed, paraffin-embedded tissue blocks. DNA was extracted from microdissected paraffin-embedded tumor sections and analyzed using a polymerase chain reaction (PCR)-based DNA sequencing method for *PIK3CA* mutations in codons [c]532-554 of exon 9 (helical domain) and c1011-1062 of exon 20 (kinase domain) [51], which included the mutation hot spot region of the *PIK3CA* proto-oncogene by Sanger sequencing following amplification of 276 bp and 198 bp amplicons, respectively. Codons 12, 13, and 61 were examined for *KRAS* and *NRAS* mutations and for *BRAF*, codons 468-474, codons 595-600, and mutations of exon 15 by pyro-sequencing were examined as previously described [52]. PTEN mutations were detected in exons 1–9 using PCR-based DNA sequencing and the lower limit of detection was approximately 20% [53]. PTEN loss by immunohistochemistry (IHC) generally indicates aberrant or mutated PTEN, which serves to activate the PI3 kinase/AKT/mTOR pathway [54, 55]. Formalin-fixed paraffin-embedded sections (5 μm thick) from biopsy or resection specimens were used for IHC analysis. The sections were stained with antibody to PTEN (Dako, Carpinteria, CA). All histology was centrally reviewed and all testing was performed in the Clinical Laboratory Improvement Amendment (CLIA) –certified Molecular Diagnostic Laboratory (MDL) at UTMDACC.

## SUPPLEMENTARY MATERIAL FIGURE AND TABLES


