# Supplementary material for: Advanced malignancies treated with a combination of the VEGF inhibitor bevacizumab, anti-EGFR antibody cetuximab, and the mTOR inhibitor temsirolimus
Source: Oncotarget. 2016 Feb 24;7(17):23227–38. doi: 10.18632/oncotarget.7594 (PMC5029622; doi:10.18632/oncotarget.7594)
Supplement: Supplementary file 1 [file oncotarget-07-23227-s001.pdf]

A Phase I Trial of Bevacizumab, Temsirolimus Alone and in Combination with Valproic Acid or Cetuximab in Patients with Advanced Malignancy and Other Indications

1.0 Objectives

1.1 Primary objective:

1.1.1 To determine the maximum tolerated doses (MTDs) and dose-limiting toxicities (DLTs) of treatment with bevacizumab and temsirolimus in combination and plus valproic acid or cetuximab.

1.2 Secondary objectives:

1.2.1 Preliminary descriptive assessment of anti-tumor efficacy of each combination

1.2.2 Preliminary assessment of the pharmacokinetic, pharmacodynamic markers of target inhibition and correlates of response (optional).

2.0 Summary: Rationale for Combining Bevacizumab, Temsirolimus with Valproic Acid or Cetuximab

Epidermal growth factor receptor (EGFR), vascular endothelial growth factor (VEGF), RET and mammalian target of rapamycin (mTOR) are validated targets in diverse malignancies. However a given tumor is unlikely to be dependent upon only one receptor or signaling pathway for its growth and survival due to the significant level of compensatory cross talk among receptors within a signaling network as well as with heterologous receptor systems. [1,2] One mechanism of tumor resistance to antiangiogenic therapy (e.g. bevacizumab) is upregulation of hypoxia-inducible factor 1α (HIF-1α), which mediates adaptive responses to hypoxic conditions.[3-11] The HIF-1-dependent activation of genes allows cancer cells to survive and metastasize in the hostile hypoxic tumor environment.[12] Increased HIF-1α is associated with increased expression VEGF, aggressive tumor growth, and poor patient prognosis.[12] HIF-1α inhibition in combination with antiangiogenic therapy is a promising strategy for targeting tumor resistance.[8,12-15] Temsirolimus has been shown to inhibit the activity of mTOR and has resulted in reduced levels of HIF-1α, HIF-2α and VEGF. The discovery of the HIF-1α inhibition properties of temsirolimus makes it an ideal candidate for combination with bevacizumab.

Furthermore histone deacetylase inhibitors (HDIs) lead to tumor regression also through antiangiogenic effects.[16] Treatment with HDIs upregulated p53 and VHL and downregulated HIF-1α and VEGF leading to inhibition of angiogenesis in in-vitro and in-vivo animal models.[16] HDIs have also been shown to inhibit VEGF-stimulated endothelial cells and angiogenesis with a greater antitumor effect observed in combination with a VEGFR tyrosine kinase inhibitor.[17]

Additionally it has been shown in preclinical models that inhibition of mTOR pathway by everolimus cooperates with EGFR inhibitors in human tumors sensitive and resistant to anti-EGFR drugs.[18] Multi-kinase targeting of the EGFR, VEGFR and mTOR may improve results with either agent alone. Thus, one attractive approach is to combine EGFR, VEGFR and mTOR inhibitors targeting downstream pathways.

Therefore combining a histone deacetylase inhibitor (valproic acid) to a VEGF inhibitor (bevacizumab) and an mTOR inhibitor (temsirolimus) targets multiple pathways in tumor progression and angiogenesis, representing a novel therapeutic approach in cancer treatment.

The next step is to determine how to combine these targeted agents safely, to establish appropriate dose and schedule for Phase II efficacy studies, and to provide preliminary data on target impact. Because these targeted agents have mostly non-overlapping toxicities, they may be amenable to escalation to full doses in combination.

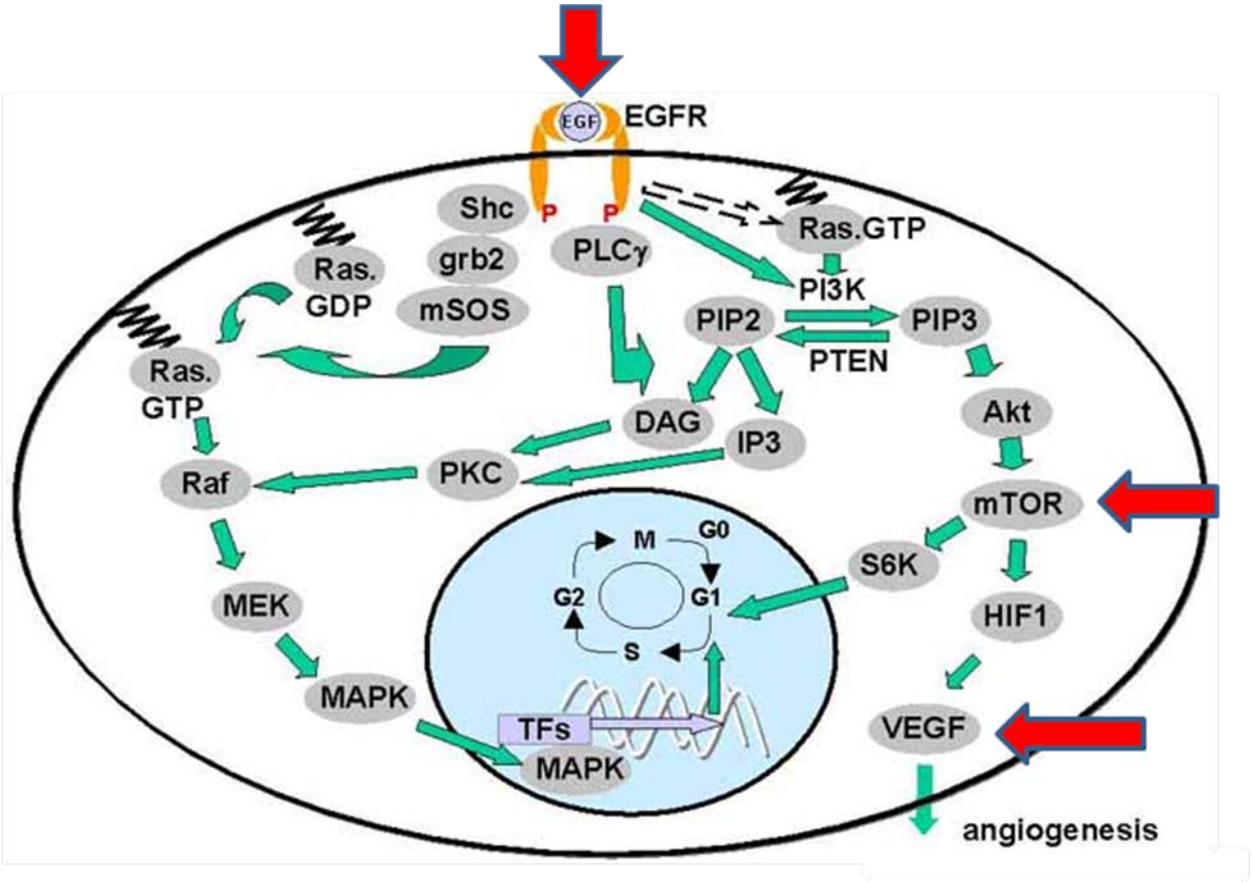

Figure 1: EGFR signaling pathway. Binding of ligand to EGFR leads to receptor dimerization, autophosphorylation and activation of downstream signaling pathways. These signals mediate cell cycle progression and angiogenesis, among other effects. Targets of cetuximab, bevacizumab and temsirolimus are shown by red arrows.

3.0 Introduction

Angiogenesis has a fundamental role in tumor growth and metastasis.[7,19,20] Antiangiogenic agents, such as the monoclonal antibody bevacizumab, target the vascular endothelial growth factor (VEGF) pathway and have demonstrated clinical benefit for a variety of malignancies, including colorectal, lung, breast, ovarian, and renal cell cancer. [6,7,21-25] Despite this progress, the biologic activity of these agents may be difficult to assess because they appear to be primarily cytostatic rather than cytotoxic, and when they are used as monotherapy, only a minority of patients have a major tumor response.[19,26] To advance the clinical testing of these agents, valid surrogate biomarkers of angiogenic activity are needed.[26] Such biomarkers would measure the agents' biological effects, determine the optimal dose, identify which patients are most likely to benefit from treatment, and monitor responses during treatment.

Dynamic contrast-enhanced magnetic resonance imaging (DCE-MRI) is a noninvasive imaging technology that can be used to measure properties of tissue microvasculature.[27] DCE-MRI is sensitive to changes in blood volume and vascular permeability that can be associated with tumor angiogenesis, and consequently DCE-MRI is a promising biomarker for characterizing tumor response to antiangiogenic treatment.[27-30] Correlative studies performed in combination with therapeutic trials have demonstrated proof of concept for DCE-MRI as a biomarker.[27]

One mechanism of tumor resistance to antiangiogenic therapy is upregulation of hypoxia-inducible factor 1α (HIF-1 α), which mediates adaptive responses to hypoxic conditions.[3-11] HIF-1α inhibition in combination with antiangiogenic therapy is a promising strategy for targeting tumor resistance.[8,12-15] Temsirolimus, a mammalian target of rapamycin (mTOR) inhibitor, has demonstrated the ability to inhibit VEGF production in vitro under both normoxic and hypoxic conditions through inhibition of hypoxia-stimulated hypoxia-inducible (HIF)-1α expression and transcriptional activation.[31] Combination treatment with bevacizumab and temsirolimus is therefore a potentially effective treatment deserving of clinical investigation.

3.1 Angiogenesis and Bevacizumab in the Treatment of Cancer

Angiogenesis, now a well-established aspect of cancer biology, is important for supplying a growing tumor with oxygen, nutrients, growth factors, hormones, proteolytic enzymes, and hemolytic factors, and is a critical step in the pathogenesis of metastasis.[7,20,32] Increased tumor vascularization and tumor expression of pro-angiogenic factors has been associated with advanced tumor stage and poor prognosis.[7] The vascular endothelial growth factor (VEGF) family of proteins and receptors play

a pivotal role in tumor angiogenesis and in the pathogenesis of a wide range of human cancers.[33] Consequently, agents that inhibit the VEGF pathway, such as bevacizumab, have generated substantial interest in the treatment of malignancy.

Bevacizumab is a recombinant, humanized, anti-VEGF monoclonal antibody developed from a murine antibody to human VEGF by recombinant DNA technology and was selected for clinical development based on preclinical evidence showing high antiangiogenic and antitumor activity.[6,34] Phase I clinical trials demonstrated that bevacizumab was relatively non-toxic and that adding bevacizumab to standard chemotherapy regimens did not significantly exacerbate chemotherapy-associated toxicities.[35] When bevacizumab was approved by the FDA as a first-line treatment for metastatic colorectal cancer in February 2004, it became the first approval by the FDA of a therapy developed to target tumor angiogenesis.[36] Further clinical trials demonstrated clinical benefit in other malignancies, including lung, breast, ovarian, and renal cell cancer.[6,7,21-25] A more detailed description of bevacizumab, including its mechanism of action, preclinical models, activity in colorectal cancer and other malignancies, and reported adverse events, is described in detail in section “Background Drug Information.”

3.2 Biomarkers for Antiangiogenic Activity

The development of angiogenesis inhibitors has underscored the need to develop new biological markers. Traditional methods of assessing response with conventional imaging may be inadequate because antiangiogenic agents appear to be primarily cytostatic rather than cytotoxic, and when they are used as monotherapy, only a minority of patients have a major tumor response.[19,26] To advance the clinical testing of antiangiogenic agents, reliable markers which can predict which patients are more likely to respond to anti-VEGF therapy are needed, but such markers remain elusive.[26,37] Such biomarkers would measure the agents’ biological effects, determine the optimal dose, identify which patients are most likely to benefit from treatment, and monitor responses during treatment.

Unfortunately, exploratory studies of the use of angiogenic factors in the serum, plasma, and urine as surrogate markers have been disappointing.[26] Measurements of VEGF, bFGF, VCAM-1, E-selectin, and matrix metalloproteinases do not consistently show correlation with clinical response.[26] The use of flow cytometry to quantify circulating endothelial cells and endothelial cell progenitors from the peripheral blood is another potential surrogate marker which is currently being studied.[19,26,37]

3.3 Dynamic Contrast-Enhanced Magnetic Resonance Imaging (DCE-MRI) as an Imaging Biomarker of Anti-angiogenic Activity

Dynamic contrast-enhanced magnetic resonance imaging (DCE-MRI) performed at high

temporal resolution following the administration of gadolinium (Gd)-chelated contrast medium is a noninvasive imaging technology that can be used to measure properties of tissue microvasculature. DCE-MRI is sensitive to changes in blood volume and vascular permeability that can be associated with tumor angiogenesis, and consequently DCE-MRI is a promising biomarker for characterizing tumor response to antiangiogenic treatment.[27-30]

The concept of an imaging biomarker is very appealing because it fulfills the need to assess tumor biology in vivo and to monitor the effects of treatment. An imaging biomarker can be measured non-invasively and repeatedly. By evaluating the entire tumor in vivo, an imaging biomarker can capture the heterogeneity of both the tumor and its response to treatment.[27-30]

DCE-MRI is performed by obtaining sequential magnetic resonance images before, during, and following the injection of gadolinium (Gd)-chelated contrast medium. The properties of the tissue microvasculature measured by DCE-MRI include:

- The volume transfer constant (K^{trans}), which is the rate of flux of contrast agent into the extracellular extravascular space within a given volume
- The extravascular volume fraction (v_e), which is the volume of the extracellular extravascular space per unit volume of tissue
- The rate constant (k_{ep}), which is the rate of backflux from the extracellular extravascular space to the vasculature.[27]

Correlative studies performed with clinical trials have demonstrated proof of concept for DCE-MRI as a biomarker.[1,27,38-40] These studies provided biological evidence of drug action by demonstrating changes in K^{trans}, k_{ep}, and/or v_e. Studies which aim to assess DCE-MRI prospectively as an early predictor of response to therapy are currently underway.

3.4 FLT-PET as an imaging biomarker of anti-tumor activity

Protocol 2008-0106 is currently IRB approved and actively enrolling patients within the Department of Investigation Cancer Therapeutics. Patients electing to participate in Dynamic Contrast Enhanced Magnetic Resonance Imaging (DCE-MRI) in this study will be eligible to participate in the the protocol 2008-0106 to evaluate if the change of FLT-PETs uptake correlates with anti-tumor response of proposed study therapy.

3.5 **Mammalian Target of Rapamycin (mTOR) Inhibition and Temsirolimus in the**

Treatment of Cancer

Temsirolimus [sirolimus 42-ester with 2,2-bis(hydroxymethyl) propionic-acid], an ester of the macrocyclic immunosuppressive agent sirolimus (rapamycin, Rapamune™), is a cytostatic cell cycle inhibitor with antitumor properties. The agent specifically inhibits the mammalian target of rapamycin (mTOR), a Ser/Thr kinase involved in the initiation of mRNA translation.[41] Temsirolimus has been shown to inhibit the growth of a wide range of histologically diverse tumor cells, with the greatest sensitivity shown by cells derived from the central nervous system (CNS) cancers, leukemia (T-cell), breast cancer, prostate cancer, and melanoma [Investigator's Brochure (Temsirolimus) 2004]. Temsirolimus is being developed as a cytostatic agent to delay the time to tumor recurrence or progression or to increase survival in patients with various malignancies. Key features of this agent include its good tolerability, unique mechanism of action, ability to arrest cells in the G_1 phase, and ability to induce apoptosis.

3.6 The Role of Hypoxia-Inducible Factor-1 (HIF-1) as a Mechanism of Resistance to Antiangiogenic Therapy

One mechanism of tumor resistance to antiangiogenic therapy is upregulation of hypoxia-inducible factor 1α (HIF-1α), which mediates adaptive responses to hypoxic conditions.[3-11] The hypoxia-mediated increase in HIF-1α is critical to the establishment and progression of many common cancers via HIF-1-dependent activation of genes that allow cancer cells to survive and metastasize in the hostile hypoxic tumor environment.[12] Increased HIF-1α is associated with increased expression VEGF, aggressive tumor growth, and poor patient prognosis.[12] HIF-1α inhibition in combination with antiangiogenic therapy is a promising strategy for targeting tumor resistance.[8,12-15]

A variety of anticancer drugs, most of which were not developed as HIF-1 inhibitors, have been reported to inhibit HIF-1.[12,15] HIF-1 inhibitors in development are currently moving toward human clinical trials.[12] One goal of HIF-1 inhibitors in clinical trials will be the ability to demonstrate that the agents can inhibit HIF-1 in patient tumors or inhibit the downstream consequences of HIF-1 inhibition such as VEGF formation. A recent preclinical DCE-MRI study evaluating the HIF-1 inhibitor PX-478 in mice bearing human xenografts demonstrated a dramatic reduction in tumor blood vessel permeability within 2 hours of treatment.[38]

3.7 Temsirolimus as an Inhibitor of Hypoxia-Inducible Factor-1 (HIF-1)

Temsirolimus is an inhibitor of mTOR. Temsirolimus binds to an intracellular protein named FKBP-12 and the protein-drug complex inhibits the activity of mTOR which controls cell division. Inhibition of mTOR activity results in G1 growth arrest in tumor cells that are treated with this agent.

When mTOR is inhibited, this blocks the ability to phosphorylate p70S6k and S6 ribosomal protein, downstream of mTOR in the PI3 kinase/AKT pathways. In *in vitro* studies with renal cell carcinoma cell lines, temsirolimus inhibited the activity of mTOR and resulted in reduced levels of HIF-1α, HIF-2α and VEGF.

3.8 **Epigenetics and Histone Deacetylase inhibitors** - Valproic acid in Cancer Treatment

The regulation of gene expression involves alterations in the primary nucleotide sequence and those that do not involve such changes in which case are called "epigenetic alterations" and include DNA methylation, Histone modifications and RNA interference.[42] Epigenetic deregulation affects several aspects of tumor cell biology and targeting these deregulated mechanisms is a promising therapeutic strategy against cancer.[42]

Histone modifications are one of the epigenetic mechanisms for regulation of gene expression. Histones are found in the nucleosomes, which are the chromatin units in which DNA is wrapped around the core histone. Chromatin can become more accessible to transcription through nucleosome remodeling and histone alterations. Histone structure can be modified through acetylation/deacetylation mechanism to produce the "histone code."[43,44] It has been found that acetylation status of the histones correlates with transcriptional activity. This "code" can be reversed and the degree of this modification correlates with gene expression.

Histone acetyl transferases (HAT) and Histone deacetylases (HDACs) are the enzymes that mediate the acetylation and deacetylation of histones. Histone acetylation is associated with increased transcription whereas histone deacetylation is associated with decreased transcription.[45]

Abnormal activity of HATs and HDACs resulting in aberrant gene transcription is commonly observed in cancer cells, especially on genes involved on cell cycle growth inhibition and differentiation which are generally repressed by HDACs.

Treatment with HDIs induces growth arrest, differentiation and apoptosis and also decreases angiogenesis presumably by inducing acetylation of histones and also of nonhistone proteins.[45,46]

Valproic acid, an antiepileptic agent, has been shown to be an effective HDI, to reduce tumor growth and metastases in animal studies. Valproic acid inhibits HDAC1 in vitro in a dose-dependent manner, with an IC50 of 0.4 mM, which is within the therapeutic range (0.35-0.7 mM in serum), for therapy in humans.[47]

4.0 Background Drug Information

4.1 Bevacizumab

Bevacizumab, a recombinant humanized monoclonal antibody, is an anti-neoplastic

agent. The drug is an IgG1 antibody that contains human framework regions and murine complementarity-determining regions. Bevacizumab binds to human vascular endothelial growth factor (VEGF) and prevents interaction of VEGF with its receptors (Flt-1, KDR) on the surface of endothelial cells. In vitro models of angiogenesis have shown that interaction of VEGF with its receptors may lead to endothelial cell proliferation and new blood vessel formation. Evidence from animal models has suggested that administration of an anti-VEGF monoclonal antibody (e.g., bevacizumab) may inhibit angiogenesis and thus may reduce microvascular growth of tumors and inhibit metastatic disease progression. Bevacizumab is metabolized and eliminated via the reticuloendothelial system. Bevacizumab has been used in different advanced solid tumors such as:

Colorectal Cancer

Bevacizumab is used in combination with IV fluorouracil-based chemotherapy for the first-line treatment of metastatic cancer of the colon or rectum. The indication for use of bevacizumab (in combination with IV fluorouracil-based chemotherapy) as initial (first-line) treatment in patients with metastatic cancer of the colon or rectum is based mainly on the results of 2 randomized, controlled clinical trials (one phase II and one phase III). In the phase III, double-blind, controlled trial, 813 patients received either a placebo or bevacizumab (5 mg/kg administered by IV infusion every 2 weeks until disease progression occurred) in conjunction with a combination irinotecan/fluorouracil/leucovorin regimen. The combination regimen consisted of irinotecan 125 mg/m^2, fluorouracil 500 mg/m^2, and leucovorin 20 mg/m^2, administered as an IV bolus injection once weekly for 4 out of every 6 weeks. Patients who received bevacizumab had a higher median overall response rate (45 versus 35%, respectively) and prolonged median overall survival (20.3 versus 15.6 months, respectively), median progression-free survival (10.6 versus 6.2 months, respectively), and median duration of response (10.4 versus 7.1 months, respectively) than patients receiving placebo (every 2 weeks) in conjunction with the same combination regimen. Grade 3 or 4 hypertension occurred more frequently (12 versus 2%) in patients receiving bevacizumab rather than placebo in conjunction with the irinotecan/fluorouracil/leucovorin regimen.

Other Uses

Bevacizumab administered in combination with carboplatin and paclitaxel is approved for the initial treatment of patients with unresectable, locally advanced, recurrent, or metastatic, nonsquamous, non-small cell lung cancer (NSCLC). A randomized, open label, multicenter clinical trial, conducted by the Eastern Cooperative Oncology Group (ECOG), in chemotherapy-naïve patients with stage IIIB/IV nonsquamous NSCLC, evaluated bevacizumab plus carboplatin and

paclitaxel versus carboplatin and paclitaxel alone and found a significant improvement in overall survival in those patients treated with bevacizumab.

Bevacizumab is being investigated for use in the treatment of breast cancer. In a phase III randomized trial, the addition of bevacizumab to capecitabine increased response rates but did not affect progression-free or overall survival in patients with previously treated metastatic breast cancer. A phase III randomized trial comparing bevacizumab with paclitaxel versus paclitaxel alone for locally recurrent or metastatic breast cancer is under way.

Bevacizumab is being investigated for use in the treatment of renal cancer. In a randomized, double-blinded, phase II trial involving 116 patients with metastatic clear-cell renal cancer, those receiving high-dose bevacizumab had longer progression-free survival than those receiving placebo. No difference in progression-free survival was observed in patients receiving low-dose bevacizumab compared with those receiving placebo, and no difference in overall survival was noted between the 3 groups. A phase III randomized trial comparing bevacizumab with interferon alfa-2b versus interferon alfa-2b alone for advanced renal cell cancer is under way.

Adverse Events Effects of Bevacizumab

Because the bevacizumab therapeutic regimen includes the use of fluorouracil and other anti-neoplastic agents, the usual cautions, precautions, and contraindications of these drugs also should be considered.

Thromboembolism. Arterial thromboembolic events, sometimes fatal, occurred at a higher incidence in patients receiving bevacizumab in combination with chemotherapy compared with those receiving chemotherapy alone. Such events included cerebral infarction, transient ischemic attacks (TIAs), myocardial infarction (MI), and angina. Deep venous thrombosis and intra-abdominal thrombosis also have been reported more frequently in patients receiving bevacizumab in combination with fluorouracil, irinotecan, and leucovorin compared with patients receiving fluorouracil, irinotecan, and leucovorin with placebo.

GI Perforations and Wound Healing Complications. GI perforation (sometimes fatal) and wound dehiscence have been reported in patients receiving bevacizumab. Bevacizumab should be permanently discontinued in patients with GI perforation or wound dehiscence requiring medical intervention.

Hemorrhage. Life-threatening, sometimes fatal, pulmonary hemorrhage has occurred in patients with non-small cell lung cancer; in a small trial, the incidence was 31% in patients with squamous cell histology and 4% in patients with adenocarcinoma receiving bevacizumab with other anti-neoplastic agents as compared with none in patients not receiving bevacizumab. Other serious

hemorrhagic effects that have been reported in patients receiving bevacizumab include GI hemorrhage, subarachnoid hemorrhage, and hemorrhagic stroke, but these were uncommon. Grade 3 or 4 gastrointestinal hemorrhage was reported in 4% of patients receiving bevacizumab with fluorouracil/leucovorin in an open-access protocol. Mild to moderate hemorrhagic events also have been reported in patients receiving bevacizumab. Grade I epistaxis was common in clinical studies and occurred in up to 35% of patients receiving bevacizumab in conjunction with other anti-neoplastic agents. If serious hemorrhage occurs, bevacizumab should be discontinued and the patient should be managed aggressively. The risk of CNS bleeding in patients with CNS metastases receiving bevacizumab has not been evaluated; patients with CNS metastases were excluded from bevacizumab studies after one such patient experienced CNS hemorrhage after receiving bevacizumab in a phase I trial.

Hypertension. Hypertension and severe hypertension have been reported in patients receiving bevacizumab. Acute increases in blood pressure have been associated with initial or subsequent infusions of bevacizumab; some cases were associated with clinical sequelae. Hypertension was more common in patients with previous history of hypertension and may respond to antihypertensive therapy. Hypertension also occurred more frequently in patients who received higher dosages (e.g., 10 mg/kg). Permanently discontinue the drug in patients who develop hypertensive crisis. Temporary suspension is recommended in patients with severe hypertension that is not controlled with medical management.

Proteinuria. Increased incidence and severity of proteinuria have been reported in patients receiving bevacizumab. In clinical trials, proteinuria ranged in severity from clinically silent to nephrotic syndrome. Discontinue the drug in patients with nephrotic syndrome. Patients receiving bevacizumab should be monitored for the development or worsening of proteinuria. The safety of continued treatment in patients with moderate to severe proteinuria has not been evaluated; such patients should be monitored regularly until improvement and/or resolution is observed. In most clinical studies, bevacizumab was interrupted for proteinuria exceeding 2 g per 24 hours and resumed when proteinuria declined below this level.

Congestive Heart Failure. Congestive heart failure has been reported in patients receiving bevacizumab. Some patients also were receiving or had previously received anthracyclines and/or left chest wall irradiation. The safety of continuation or resumption of bevacizumab in patients who develop cardiac dysfunction has not been studied.

Infusion Reactions. In clinical studies, infusion reactions with the first bevacizumab dose were uncommon (less than 3%). Severe infusion reactions occurred in 0.2% of patients receiving

bevacizumab. Infusion reactions include hypertension, hypertensive crises associated with neurologic manifestations, wheezing, oxygen desaturation, grade 3 hypersensitivity, chest pain, headaches, rigors, and diaphoresis.

4.2 **Temsirolimus**

The observed antitumor and immunosuppressive properties of rapamycin analogs are due to their ability to disrupt the mTOR-dependent signaling pathway.[48] mTOR, a member of the phosphatidylinositide 3'-kinase (PI3K)-related family, is located predominantly in the nuclear fraction of both neoplastic and normal cells.[49] mTOR activation triggers resting cells to increase the translation of a subset of mRNAs whose proteins are required for cell cycle progression from G_1 to S phase. mTOR regulates essential signal transduction pathways and is involved in the coupling of growth stimuli with cell cycle progression. Experimental data indicate that mTOR acts downstream of the PI3K/Akt pathway and is phosphorylated in response to mitogenic signals.[48] Early studies reported that mTOR was dedicated to initiating mRNA translation in response to favorable nutrient environments.[50] In fact, cells treated with rapamycin undergo changes that are strikingly similar to those observed during conditions of starvation. These include mTOR inactivation, down regulation of translation, G_1 arrest, accumulation of glycogen stores and altered transcription patterns.[50] More recent studies have demonstrated that mTOR is involved in regulating many aspects of cell growth, including organization of the actin cytoskeleton, membrane traffic, protein degradation, protein kinase C (PKC) signaling, ribosome biogenesis, and transcription.[51]

Temsirolimus reacts with the ubiquitous intracellular FK506-binding protein 12 (FKBP12), forming a Temsirolimus/FKBP12 complex that is a potent inhibitor of the highly conserved kinase mTOR.[52] Inhibition of mTOR leads to suppression of several downstream signaling effectors, including the ribosomal subunit $p70^{S6k}$ and the eukaryotic initiation factor 4 binding protein 1 (4E-BP1).[53] These two proteins play key roles in ribosomal biogenesis and cap-dependent translation, respectively.[54] The extent of phosphorylation of these two downstream proteins ($p70^{S6}$ kinase and 4E-BP1) may therefore serve as indicators of temsirolimus biologic activity in vivo. Inhibition of the synthesis of ribosomal proteins and elongation factors, required to accelerate the process of cell division, are thought to contribute to the anti-proliferative effects of rapamycin analogs.[55] While temsirolimus inhibits the translation of only a subset of mRNAs, inhibition of mTOR can lead to a substantial decrease (~15%) in overall protein synthesis.[56]

Pharmacokinetics. Cytochrome P450 3A4 is the major isozyme responsible for the formation of five Temsirolimus metabolites. Sirolimus, an active metabolite of temsirolimus, is the principal metabolite in humans following intravenous treatment. The remainder of the metabolites

account for less than 10% of radioactivity in the plasma. In human liver microsomes temsirolimus was an inhibitor of CYP2D6 and 3A4. There was no effect observed in vivo when temsirolimus was administered with desipramine (a CYP2D6 substrate), and no effect is anticipated with substrates of CYP3A4 metabolism. Temsirolimus is cleared predominately by the liver and elimination of the drug is primarily via the feces.

Uses. Temsirolimus is used as a single-agent for the treatment of advanced **renal cell carcinoma** in patients. The current indication for temsirolimus is based principally on the results of a multi-center, randomized, open label clinical trial involving 626 patients with poor prognosis, previously untreated, metastatic renal-cell carcinoma.[57] Patients in this study needed at least three of six predictors of short term survival. These predictors included: a serum lactate dehydrogenase level of more than 1.5 times the upper limit of normal (ULN) range, a hemoglobin level below the lower limit of the normal range, a corrected serum calcium level of more than 10 mg/dL, a time from initial diagnosis of renal cell carcinoma to randomization of less than one year, a Karnofsky performance score of 60-70, or metastases in multiple organs. Prior to therapy, patients were stratified for prior nephrectomy status within three geographic regions and were randomly assigned (1:1:1) to receive IFN-α alone (n=207), temsirolimus alone (25mg weekly; n=209), or the combination arm (n=210). The objectives of this study were to compare Overall Survival (OS), Progression-Free Survival (PFS), Objective Response Rate (ORR), and safety in patients receiving IFN-α to those receiving temsirolimus or temsirolimus plus IFN-α. Patient demographics were comparable between the three treatment arms with regards to age, gender, and race. The median age of all groups was 59 (range 23-86). Sixty-nine percent of patients were male and 31% were female. The racial distribution for all groups was 91% White, 4% Black, 2% Asian, and 3% other. Sixty-seven percent of patients had a history of prior nephrectomy. The median duration of treatment in the temsirolimus arm was 17 weeks (range 1-126 weeks). The median duration of treatment on the IFN-α was 8 weeks (range 1-124 weeks). Patients who received temsirolimus alone had longer overall survival (hazard ratio for death, 0.73; 95% confidence interval [CI], 0.58 to 0.92; P=0.008) and progression-free survival (P<0.001) than did patients who received interferon alone. Overall survival in the combination-therapy group did not differ significantly from that in the interferon group (hazard ratio, 0.96; 95% CI, 0.76 to 1.20; P=0.70). Median overall survival times in the interferon group, the temsirolimus group, and the combination-therapy group were 7.3, 10.9, and 8.4 months, respectively.

Temsirolimus safety, pharmacokinetics, and preliminary antitumor effects were evaluated in a phase I dose-escalation study with doses of 7.5-220 mg/m^2 given as a weekly intravenous (IV) infusion to 24 patients with advanced malignancies.[58] Although the maximum tolerated dose (MTD)

was not reached, 220 mg/m^2 appeared to be the maximum acceptable dose, with thrombocytopenia being dose limiting with repeated dosing. No clinically relevant immunosuppressive effects were observed during treatment, although herpes simplex infections were observed in five patients. The most frequent drug-related adverse events were acneiform maculopapular rashes and mucositis/stomatitis (18 of 24 patients, 75%). Confirmed partial responses (PRs) were observed in 2 of 24 patients evaluated, one each with RCC and breast cancer.

Data from *in vitro* studies of A498 human renal cell lines indicated that temsirolimus had a median growth inhibitory concentration (IC_{50}) of 5 ng/mL [Investigator's Brochure (CCI-779) 2004]. Predicted modeling of IC_{50} (humans receiving doses as low as 10 mg) suggests that whole blood concentrations would be above the range of 1 ng/mL throughout the entire 1-week dose interval and above 5 ng/mL for the majority of this time period. It is expected that mTOR inhibition will be attained with a 25 mg dose.

Clinical pharmacokinetic data are available in patients with cancer receiving temsirolimus both IV daily x 5 days every 2 weeks, once weekly schedules, and orally daily × 5 days every 2 weeks. These data indicate that there is no appreciable drug accumulation between cycles and that distribution is extensive. With increasing dose, exposure (AUC) increases in a less than proportional fashion. The mean volume of distribution at steady state (V_{dss}) is large (57 L after 2 mg IV dose; 900 L following a 250 mg IV dose) and increases with dose. Exposure to the hydrolytic product sirolimus is substantial with mean values of approximately 1.5-2.3-fold greater than those seen with temsirolimus following IV administration. Clearance (CL) of temsirolimus from whole blood increases with increasing dose from approximately 5.2 L/h after a 2 mg dose to 100 L/h after a 250 mg dose. Intersubject variability in CL at a given dose was modest and ranged from 16-27%. The terminal half-life ($t_{1/2}$) following temsirolimus doses of 25 to 250 mg is approximately 15 hours.

Pharmacokinetic results from the initial phase I study showed that the AUC increased proportionally with doses up to 150 mg, but doses higher than 300 mg yielded high AUCs and low CL in some patients.[58] The mean V_{dss} was large with mean values of 127-384 L, while the temsirolimus mean terminal $t_{1/2}$ decreased from 22 hours (34 mg/m^2) to 13 hours (220 mg/m^2) as the dose increased. Similar pharmacokinetic data were reported for 16 patients following their initial dose of temsirolimus of 25, 75, or 250 mg delivered as a weekly 30-minute IV infusion.[59]

To better characterize the relationship between dose and tolerability of temsirolimus, a phase II study of the agent in patients with advanced refractory RCC randomly assigned 111 patients to flat doses of 25, 75, or 250 mg IV weekly.[59] Overall, there was 1 complete response, 7 PRs, and 29 minor responses, but results for each dose level group were similar with respect to both toxicity and

efficacy. This observation, together with the greater number of dose reductions and treatment discontinuations at the higher dose levels led investigators to recommend 25 mg IV weekly as the optimum dose level for further temsirolimus studies. However, a study of temsirolimus in patients with malignant glioma receiving enzyme-inducing antiepileptic drugs (EIAEDs) reported that 250 mg IV weekly was the recommended phase II dose and MTD of the agent in that population.[60] In the phase II trial reported by Atkins *et al*. (2004), patients were retrospectively assigned to risk categories (good, intermediate, or poor) based on factors described by Motzer *et al*. (2002), and median survival data were compared to historic data for interferon-α treatment. Temsirolimus-treated patients in the intermediate- and poor-risk groups had longer median survival (19.3 and 8.2 months, respectively) than intermediate and poor-risk groups treated with interferon-α (13.8 and 4.9 months, respectively), suggesting that mTOR inhibition may be of particular relevance in poor prognosis RCC.[59]

A phase II study has been reported in patients with metastatic melanoma.[61] 33 patients were treated with temsirolimus, 250 mg IV weekly. Only one patient had a partial response, and median time to progression was 10 weeks.

Adverse Effects of Temsirolimus

In a randomized, open label phase III of interferon alfa alone, temsirolimus alone, and temsirolimus and interferon alfa, a total of 616 patients were treated. Of these patients, 208 received Temsirolimus 25mg weekly. The most common adverse reactions that occurred with a frequency greater than 30 percent were rash, asthenia, mucositis, nausea, edema, and anorexia. The most common laboratory abnormalities that occurred with a frequency of greater than 30 percent were anemia, hyperglycemia, hyperlipemia, hypertriglyceridemia, elevated serum alkaline phosphatase, elevated serum creatinine, lymphopenia, hypophosphatemia, thrombocytopenia, elevated serum aspartate transaminase (AST), and leukopenia.

Severe adverse reactions (grade 3 or 4) included asthenia, dyspnea, rash, and pain. **Rare** serious adverse reactions associated with temsirolimus included interstitial lung disease, bowel perforation, and acute renal failure. Severe laboratory abnormalities (grade 3 or 4) included hypertriglyceridemia, anemia, hypophosphatemia, hyperglycemia, lymphopenia, and neutropenia.

Hyperglycemia/Glucose Intolerance. In the phase III trial, 89% of patients receiving temsirolimus had at least one elevated serum glucose while on treatment, and 26% of patients reported hyperglycemia as an adverse event. This may result in the need for an increase in the dose of, or initiation of insulin and/or oral hypoglycemic therapy. Serum glucose should be tested before and during treatment. Patients should be advised to report excessive thirst or any increase

in the volume of urination.

Infections. Temsirolimus may result in immunosuppression. Patients should be carefully observed for the occurrence of infections, including opportunistic infections.

Intersitial Lung Disease. Cases of interstitial lung disease, some resulting in death, occurred in patients who received temsirolimus. Some patients were asymptomatic with infiltrates detected on computed tomography scan or chest radiograph. Others presented with symptoms such as dyspnea, cough, hypoxia, and fever. Some patients required discontinuation of temsirolimus and/or other treatment with corticosteroids and/or antibiotics, while some patients continued with treatment without additional intervention. Patients should be advised to report promptly any new or worsening respiratory symptoms.

Hyperlipidemia. The use of temsirolimus is likely to result in increases in serum triglycerides and cholesterol. In the phase III trial, 87% of patients receiving temsirolimus had at least one elevated serum cholesterol value and 83% had at least one elevated serum triglyceride value. This may require initiation, or increase in the dose, of lipid-lowering agents. Serum cholesterol and triglycerides should be tested before and during treatment with temsirolimus.

Bowel Perforation. Cases of fatal bowel perforation occurred in patients who received temsirolimus. These patients presented with fever, abdominal pain, metabolic acidosis, bloody stools, diarrhea, and/or acute abdomen. Patients should be advised to report promptly any new or worsening abdominal pain or blood in their stools.

Renal Failure. Cases of rapidly progressive and sometimes fatal acute renal failure not clearly related to disease progression occurred in patients who received temsirolimus. Some of these cases were not responsive to dialysis.

Wound Healing Complications. Use of temsirolimus has been associated with abnormal wound healing. Therefore, caution should be exercised with the use of temsirolimus in the perioperative period.

Intracerebral Hemorrhage. Patients with central nervous system tumors (primary CNS tumor or metastasis) and/or receiving anticoagulation therapy may be at an increased risk of developing intracerebral bleeding (including fatal outcomes) while receiving temsirolimus.

Co-administration with Inducers or Inhibitors of CYP3A Metabolism.

Agents Inducing CYP3A Metabolism:

Strong inducers of CYP3A4/5 such as dexamethasone, carbamazepine, phenytoin, phenobarbital, rifampin, rifabutin, and rifampacin may decrease exposure of the active metabolite, sirolimus. If

alternative treatment can not be administered, a dose adjustment should be considered. St. John's Wort may decrease temsirolimus plasma concentrations unpredictably. Patients receiving temsirolimus should not take St. John's Wort concomitantly.

Agents Inhibiting CYP3A Metabolism:

Strong CYP3A4 inhibitors such as atazanavir, clarithromycin, indinavir, itraconazole, ketoconazole, nefazadone, nelfinavir, ritonavir, saquinavir, and telithromycin may increase blood concentrations of the active metabolite sirolimus. If alternative treatments can not be administered, a dose adjustment should be considered.

Vaccinations. The use of live vaccines and close contact with those who have received live vaccines should be avoided during treatment with temsirolimus. Examples of live vaccines are: intranasal influenza, measles, mumps, rubella, oral polio, BCG, yellow fever, varicella, and TY21a typhoid vaccines.

Pregnancy. Temsirolimus is Pregnancy Category D. Temsirolimus administered daily as an oral formulation caused embryo-fetal and intrauterine toxicities in rats and rabbits at human sub-therapeutic exposures. Embryo-fetal adverse effects in rats consisted of reduced fetal weight and reduced ossifications, and in rabbits included reduced fetal weight, omphalocele, bifurcated sternabrae, notched ribs, and incomplete ossifications.

In rats, the intrauterine and embryo-fetal adverse effects were observed at the oral dose of 2.7mg/m^2/day (approximately 0.04-fold the AUC in cancer patients at the human recommended dose). In rabbits, the intrauterine and embryo-fetal adverse effects were observed a the oral dose of >= 7.2mg/m^2/day (approximately 0.12-fold the AUC in cancer patients at the recommended human dose).

Women of childbearing potential should be advised to avoid becoming pregnant throughout treatment and for 3 months after temsirolimus therapy has stopped. Temsirolimus can cause fetal harm when administered to a pregnant woman. If this drug is used during pregnancy, or if the patient becomes pregnant while taking this drug, the patient should be apprised of the potential hazard to the fetus.

Men should be counseled regarding the effects of temsirolimus on the fetus and sperm prior to starting treatment. In male rats, the following fertility effects were observed: decreased number of pregnancies, decreased sperm concentration and motility, decreased reproductive organ weight, and testicular tubular degeneration. These effects were observed at oral temsirolimus doses >= 3mg/m^2/day (approximately 0.2-fold the human recommended intravenous dose). Fertility was absent

at 30mg/m^2/day. Men with partners of childbearing potential should use reliable contraception throughout treatment and are recommended to continue this for 3 months after the last dose of temsirolimus.

4.3 Cetuximab

Cetuximab, a recombinant chimeric (human-murine) monoclonal antibody, is an antineoplastic agent. The drug is an immunoglobulin containing human framework (i.e., IgG1 heavy and kappa light constant regions) and murine Fv regions. Cetuximab binds specifically to the extracellular domain of the human epidermal growth factor receptor (EGFR, HER1, c-ERbB-1) on both normal and tumor cells and competitively blocks the cellular action of EGF and other ligands (e.g., transforming growth factor [TGF]-a). EGFR is a transmembrane glycoprotein that belongs to the subfamily of type I receptor tyrosine kinases, which include EGFR (HER1), HER2, HER3, and HER4. While EGFR is expressed in many normal epithelial tissues (e.g., skin, hair follicle), overexpression of the glycoprotein is detected in human carcinomas (e.g., colon, rectum). Binding of cetuximab to EGFR blocks phosphorylation and activation of receptor-associated kinases resulting in inhibition of cell growth, induction of apoptosis (programmed cell death), and decreased matrix metalloproteinase and vascular endothelial growth factor production.

Evidence from in vitro tests and in vivo animal studies has suggested that cetuximab may inhibit growth and survival of tumor cells that overexpress EGFR, while such antitumor effects were not observed in human cancer xenografts that lacked the EGFR expression. In animal studies, addition of cetuximab to irinotecan or to a combination chemotherapy regimen of irinotecan and fluorouracil resulted in an increased antitumor effect when compared with chemotherapy alone. Following administration of the recommended regimen of cetuximab (initial loading dose, followed by weekly maintenance doses), steady state cetuximab concentrations are achieved by the third weekly infusion; the mean half-life of cetuximab following multiple dosing is 114 hours. The major route of clearance from the circulation is believed to be through internalization of the cetuximab EGFR complex on hepatocytes and skin.

Cetuximab has been used in the following cancers:

Colorectal Cancer. Cetuximab is used in combination with irinotecan for the treatment of metastatic colorectal cancer that is refractory to irinotecan-based chemotherapy in patients with tumors that express the epidermal growth factor receptor (EGFR). Cetuximab also is used as a single

agent for the treatment of EGFR-expressing, metastatic colorectal cancer in patients who are intolerant of irinotecan-based chemotherapy. The current indications are based on objective response rates; there currently are no data demonstrating a clinical benefit (e.g., improvement in disease-related symptoms, increased survival).

Head and Neck Cancer. Cetuximab has been designated an orphan drug by the US Food and Drug Administration (FDA) for the treatment of squamous cell cancer of the head and neck in patients who express EGFR. The drug is being investigated in combination with radiation therapy or other antineoplastic agents (e.g., cisplatin) for this use.

Adverse Effects of Cetuximab

Infusion-related Effects. Mild or moderate (grade 1 or 2) infusion-related effects (e.g., chills, fever, dyspnea) have been reported in 16% of patients receiving cetuximab in combination with irinotecan and in 19% of those receiving cetuximab monotherapy. Severe infusion-related effects (e.g., rapid airway obstruction [bronchospasm, stridor, hoarseness], urticaria, hypotension), which rarely may be fatal, have been reported in 3% of patients receiving cetuximab infusion. Approximately 90% of severe infusion reactions occurred in association with the initial infusion of cetuximab despite premedication with antihistamines; grade 1 or 2 infusion reactions were usually observed on the first day of initial dosing. However, the first severe infusion reaction may not occur until subsequent infusions, and caution must be exercised with *every* cetuximab infusion. Patients should be monitored during and for 1 hour following cetuximab infusion for signs of infusion reaction. For patients experiencing infusion reactions, longer observation periods may be required. In clinical studies, mild or moderate infusion-related effects were managed by reducing the infusion rate and administering antihistamines (e.g., diphenhydramine) prior to subsequent doses. If severe infusion-related effects occur, cetuximab therapy should be immediately and permanently discontinued, and appropriate therapy (e.g., epinephrine, corticosteroids, IV antihistamines, bronchodilators, oxygen) initiated. Patients should be carefully observed until all infusion-related manifestations have completely resolved.

Pulmonary Effects. Interstitial lung disease, interstitial pneumonitis (fatal in one case), and exacerbation of preexisting fibrotic lung disease have been reported in patients receiving cetuximab alone or in combination with other antineoplastic agents (e.g., cisplatin, irinotecan). These adverse pulmonary effects generally occurred between the fourth and eleventh doses of cetuximab. If acute onset or exacerbation of pulmonary manifestations occurs, cetuximab therapy should be interrupted,

and these manifestations should be promptly investigated. If interstitial lung disease is confirmed, cetuximab should be discontinued, and appropriate therapy instituted.

Electrolyte Effects. Electrolyte abnormalities, sometimes severe, including hypomagnesemia, hypocalcemia, and hypokalemia, have occurred in patients receiving cetuximab. Interim analysis of data for 244 patients in controlled clinical trials shows that the incidence of overall or severe (grade 3 or 4) hypomagnesemia was increased in patients receiving cetuximab (alone or in combination with chemotherapy) compared with those receiving best supportive care or chemotherapy alone. About half of patients receiving cetuximab experienced hypomagnesemia, and 10-15% experienced severe hypomagnesemia. The onset of electrolyte abnormalities may occur from days to months following initiation of cetuximab therapy. Electrolyte repletion therapy should be administered as necessary and, in severe cases, intravenous replacement therapy is required. Because the time to resolution of electrolyte abnormalities is not established, continued monitoring following completion of cetuximab therapy is recommended.

Dermatologic Effects. Acneiform rash (e.g., multiple follicular- or pustular-appearing lesions) has been reported in 89% of patients receiving cetuximab in clinical studies; severe (grade 3 or 4) acneiform rash has been reported in 11% of patients. Acneiform rash occurred most frequently on the face, upper chest, and back but could extend to the extremities. Acneiform rash generally appears within the first 2 weeks of cetuximab therapy and may resolve following discontinuance of cetuximab; however, manifestations have persisted beyond 28 days in nearly 50% of cases. Other adverse dermatologic effects, including skin drying/fissuring and inflammation (e.g., blepharitis, cheilitis, cellulitis, cyst), also have been reported. Adverse dermatologic effects may result in infectious complications;*Staphylococcus aureus* sepsis and abscesses requiring incision and drainage have been reported in patients receiving cetuximab in clinical studies.

Patients who develop severe acneiform rash should receive reduced cetuximab dosages. Patients who experience adverse dermatologic effects while receiving cetuximab should be monitored for development of inflammatory or infectious complications, and appropriate therapy instituted. Treatment with topical and/or oral antibiotics should be considered; topical corticosteroids are not recommended.

Serious Adverse Effects. Other serious adverse effects associated with cetuximab include diarrhea (6% in patients also receiving irinotecan; 0.2% in patients receiving cetuximab monotherapy), dehydration (5 or 2% of patients receiving cetuximab with irinotecan or cetuximab monotherapy, respectively), fever (5%), sepsis (3%), and renal failure (2%).

Nail Disorder. Paronychial inflammation (particularly of the great toes and thumbs) has been reported in 14% of patients receiving cetuximab.

EGFR Testing. Assessment for epidermal growth factor receptor (EGFR) expression should be performed by laboratories with demonstrated proficiency in the specific technology being utilized. Improper assay performance, including use of suboptimally fixed tissue, failure to utilize specified reagents, deviation from specific assay instructions, and failure to include appropriate controls for assay validation, may lead to unreliable results.

Therapy Monitoring. Patients should be monitored periodically for hypomagnesemia, and accompanying hypocalcemia and hypokalemia, during and following the completion of cetuximab therapy. Monitoring should be continued for at least 8 weeks following completion of therapy, which corresponds to the observed half-life and persistence of cetuximab.

Immunologic Effects. Non-neutralizing anticetuximab antibodies were detected in about 5% of patients (28/530) receiving cetuximab; the median time to onset was 44 days. Although the incidence of antibody development has not been established, there appears to be no relationship between the appearance of antibodies to cetuximab and the safety and efficacy of the drug.

4.4 Valproic Acid

Valproic acid is administered orally. It is FDA approved for treatment of seizure, mood stabilization in bipolar disorder, and migraine prophylaxis. Valproic acid is FDA approved for seizure, bipolar disorder and migraine prophylaxis. It is commercially available and will be prescribed for patients.
Reported adverse events and potential risks:

COMMON

>10%:

Central nervous system: Headache (≤31%), somnolence (≤30%), dizziness (12% to 25%), insomnia (>1% to 15%), nervousness (>1% to 11%), pain (1% to 11%)

Dermatologic: Alopecia (>1% to 24%)

Gastrointestinal: Nausea (15% to 48%), vomiting (7% to 27%), diarrhea (7% to 23%), abdominal pain (7% to 23%), dyspepsia (7% to 23%), anorexia (>1% to 12%)

Hematologic: Thrombocytopenia (1% to 24%; dose related)

Neuromuscular & skeletal: Tremor (≤57%), weakness (6% to 27%)

Ocular: Diplopia (>1% to 16%), amblyopia/blurred vision (≤12%)

Miscellaneous: Infection (≤20%), flu-like syndrome (12%)

5.0 Patient Eligibility

5.1 **Inclusion:**

5.1.1 Patients with advanced or metastatic cancer that is refractory to standard therapy, relapsed after standard therapy, or have no standard therapy that induces a complete response of at least 10% or improves survival by at least three months. In addition, patients with diseases that are "benign" by pathology, but relentlessly progressive, leading to disability, pain, and premature death in the majority of cases (including but not limited to lymphangioleiomyomatosis (LAM), type 2 neurofibromatosis (NF), Erdheim Chester disease, and Castleman's disease) may also be considered for enrollment

5.1.2 Patients should be at least four weeks from the last day of therapeutic radiation or cytotoxic chemotherapy or from antibody therapy, or at least five half-lives from non-cytotoxic targeted or biologic therapy. Patients may have received palliative radiation immediately before (or during) treatment provided radiation is not to the only target lesion available.

5.1.3 ECOG performance status </= 2 (Karnofsky >/= 60%).

5.1.4 Lansky performance status of >/= 60% for participants 16 years old or younger.

5.1.5 Patients must have allowable organ and marrow function defined as: absolute neutrophil count >/= 1,000/mL; platelets >/=50,000/mL; creatinine </= 3 X ULN; total bilirubin </= 3.0; AST(SGOT)/ALT(SGPT) </= 5 X ULN; fasting level of total cholesterol of no more than 350mg/dL; triglyceride level of no more than 400mg/dL.

5.1.6 Women of child-bearing potential and men must agree to use adequate contraception (hormonal or barrier method of birth control; abstinence) prior to study entry, for the duration of study participation, and for 90 days after the last dose.

5.1.7 Ability to understand and the willingness to sign a written informed consent document.

5.1.8 Patients may not be receiving any other investigational agents and/or any other concurrent anticancer agents or therapies.

5.2 Exclusion:

5.2.1 Patients with clinically significant unexplained bleeding within 28 days prior to entering the study.

5.2.2 Uncontrolled systemic vascular hypertension (Systolic blood pressure > 140

mmHg, diastolic blood pressure > 90 mmHg on medication).

5.2.3 Patients with clinically significant cardiovascular disease: History of CVA within 6 months, Myocardial infarction or unstable angina within 6 months, Unstable angina pectoris

5.2.4 Pregnant or breast-feeding women.

5.2.5 History of hypersensitivity to bevacizumab, murine products, or any component of the formulation.

5.2.6 History of hypersensitivity to Temsirolimus or its metabolites (including sirolimus), polysorbate 80, or to any component of the formulation.

5.2.7 History of hypersensitivity to cetuximab, murine products, or any component of the formulation.

5.2.8 Patients that are taking CYP3A4 inducers and/or inhibitors. Please see section "Drug Information" for details. If a patient has a history of taking CYP3A4 inducers and/or inhibitors prior to enrollment on the protocol, it is strongly recommended that the patient stops the drug and waits at least 5 half-lives of said drug before initiating therapy on protocol.

5.2.9 Colorectal cancer patients with known KRAS mutation (for the arm combining bevacizumab, temsirolimus and cetuximab)

5.2.10 Patients who have had major surgery within 6 weeks of enrollment in the study.

6.0 Treatment Plan

6.1 As two or more drugs being given in combination are being tested in this protocol, there is a need to explore a variety of dose levels.

6.2 A MTD is defined as the dose level below the dose at which 2 of 6 patients experience drug-related dose limiting toxicity (DLT) in the first cycle.

6.3 Dose escalation will proceed as described below in Table 1 through Table 3 depending on the arm on which the patient is enrolled.

6.3.1 Whenever a dose level is determined to be above the MTD, dose escalation will halt. Three additional patients will be entered at the next lowest dose level if only 3 patients were treated previously at that dose.

6.4 This protocol will utilize a standard 3+3 Phase I escalation design with three patients per cohort in an effort to obtain three evaluable patients. Three patients will be entered

at each dose level in order to obtain adequate correlative data in addition to the safety data. Three patients will be treated per dose level and evaluated for toxicity. Dose escalation will then proceed as follows:

**Number of Patients with DLT at a Given Dose Level**	**Escalation Decision Rule**
0 out of 3	Enter 3 patients at the next dose level.
≥2 out of 3	Dose escalation will be stopped. Three (3) additional patients will be entered at the next lowest dose level if only 3 patients were treated previously at that dose.
1 out of 3	Enter 3 more patients at this dose level.<br>• If 0 of these 3 patients experience DLT, proceed to the next dose level.<br>• If 1 or more of this group suffer DLT, then dose escalation is stopped, and next lowest dose is declared as a maximum tolerated dose (MTD).<br>• If 2 or more of this group suffer DLT, then dose escalation is stopped. Three (3) additional patients will be entered at the next lowest dose level if only 3 patients were treated previously at that dose.

6.5 The MTDs identified will be expanded by up to 10 additional patients to further evaluate toxicity and correlative data.

6.6 There will be no intra-patient dose escalation, and no patients will be enrolled in the next dose level until three patients enrolled at the previous dose level have completed at least four weeks of therapy. If a DLT is observed in one of the three patients after one cycle, then dose escalation will not proceed until six patients in the cohort have

been assessed for toxicity after one cycle.

6.7 Patients will continue treatment until their disease worsens, their side effects become too severe, or the patient's physician feels it is not in the patient's best interest to continue. A patient may also be discontinued for an intercurrent illness that prevents further administration of treatment. A patient may also choose to discontinue enrollment in the protocol at any time.

6.8 Pre-medication, precautions, route, and schedule for each medication are described in Table "Dosing Regimen."

6.9 If a response has been observed in a particular tumor type with the study drug or drug combination, then the study may be expanded to include a total of 14 participants with that tumor type. All patients will be treated at the highest current dose level. All enrolled participants will be considered in the DLT analysis. If at any time more than or equal to one third of the participants at a dose level experience DLT, that dose is considered to be above the MTD. For the purpose of adding up to 14 additional participants, a tumor response is defined as one or more of the following: (1) stable disease for more than or equal to 4 months, (2) decrease in measurable tumor (sentinel lesions) by more than or equal to 20% by RECIST criteria, (3) decrease in tumor markers by more than or equal to 25% (for example, a >/= 25% decrease in CA125 for patients with ovarian cancer), or (4) a partial response according to the Choi criteria, i.e. decrease in size by 10% or more, or a decrease in the tumor density, as measured in Hounsfield units (HU), by more than or equal to 15% (28).

6.10 Up to 3 additional patients may be added to a cohort for evaluation of correlative studies. These patients will be considered in the DLT analysis.

Patients will be assigned to the 3 different treatment combinations mentioned below at the discretion of the treating physician.

TABLE 1: Dose-Escalation Schedule for Bevacizumab/Temsirolimus AND Cetuximab			
Dose Level	Dose and Schedule (28-day cycle)		
	Temsirolimus*	Bevacizumab	Cetuximab
Level -1	5 mg days 1, 8, 15 and 22	2.5 mg/kg day 1 and 15	Loading 75 mg/m² Maintenance 50 mg/m² days 1, 8, 15 and 22
Level 0	5 mg days 1, 8, 15 and 22	2.5 mg/kg day 1 and 15	Loading 100 mg/m² Maintenance 75 mg/m² days 1, 8, 15 and 22
Level 1	5 mg days 1, 8, 15 and 22	5 mg/kg day 1 and 15	Loading 100 mg/m² Maintenance 75 mg/m² days 1, 8, 15 and 22
Level 2	5 mg days 1, 8, 15 and 22	10 mg/kg day 1 and 15	Loading 100 mg/m² Maintenance 75 mg/m² days 1, 8, 15 and 22
Level 3	12.5 mg days 1, 8, 15 and 22	2.5 mg/kg day 1 and 15	Loading 100 mg/m² Maintenance 75 mg/m² days 1, 8, 15 and 22
Level 4	12.5 mg days 1, 8, 15 and 22	7.5 mg/kg day 1 and 15	Loading 200 mg/m² Maintenance 75 mg/m² days 1, 8, 15 and 22
Level 5	12.5 mg days 1, 8, 15 and 22	10 mg/kg day 1 and 15	Loading 200 mg/m² Maintenance 125 mg/m² days 1, 8, 15 and 22
Level 6	20 mg days 1, 8, 15 and 22	2.5 mg/kg day 1 and 15	Loading 200 mg/m² Maintenance 125 mg/m² days 1, 8, 15 and 22
Level 7	20 mg days 1, 8, 15 and 22	7.5 mg/kg day 1 and 15	Loading 200 mg/m² Maintenance 125 mg/m² days 1, 8, 15 and 22
Level 8	20 mg days 1, 8, 15 and 22	10 mg/kg day 1 and 15	Loading 200 mg/m² Maintenance 125 mg/m² days 1, 8, 15 and 22
Level 9	25 mg days 1, 8, 15 and 22	2.5 mg/kg day 1 and 15	Loading 400 mg/m² Maintenance 250 mg/m² days 1, 8, 15 and 22
Level 10	25 mg days 1, 8, 15 and 22	5 mg/kg day 1 and 15	Loading 400 mg/m² Maintenance 250 mg/m² days 1, 8, 15 and 22
Level 11	25 mg days 1, 8, 15 and 22	10 mg/kg day 1 and 15	Loading 400 mg/m² Maintenance 250 mg/m² days 1, 8, 15 and 22

* ***Temsirolimus dosing for the pediatric participants:*** If participants are 40 kg or greater, then the dosing can

be the same as for adults. However if <40kg, then at the 5 mg adult dose level, <40kg children be given 0.1 mg/kg temsirolimus; at the 12.5 mg adult dose level, children <40kg get 0.25 mg/kg; and at the 20 mg temsirolimus adult dose level, children <40kg receive 0.4 mg/kg temsirolimus.

TABLE 2: Dose-Escalation Schedule for Bevacizumab/Temsirolimus AND Valproic Acid

Dose Level	Dose and Schedule (28-day cycle)		
	**Temsirolimus***	**Bevacizumab**	**Valproic acid**
Level -1	5 mg days 1, 8, 15,and 22	2.5 mg/kg day 1 and 15	5 mg/kg rounded to nearest 250mg daily on days 1-7 and 15-21
Level 0	5 mg days 1, 8, 15 and 22	5 mg/kg day 1 and 15	5 mg/kg rounded to nearest 250mg daily on days 1-7 and 15-21
Level 1	5 mg days 1, 8, 15 and 22	10 mg/kg day 1 and 15	5 mg/kg rounded to nearest 250mg daily on days 1-7 and 15-21
Level 2	12.5 mg days 1, 8, 15 and 22	2.5 mg/kg day 1 and 15	5 mg/kg rounded to nearest 250mg daily on days 1-7 and 15-21
Level 3	12.5 mg days 1, 8, 15 and 22	7.5 mg/kg day 1 and 15	10 mg/kg rounded to nearest 250mg daily on days 1-7 and 15-21
Level 4	12.5 mg days 1, 8, 15 and 22	10 mg/kg day 1 and 15	10 mg/kg rounded to nearest 250mg daily on days 1-7 and 15-21
Level 5	20 mg days 1, 8, 15 and 22	2.5 mg/kg day 1 and 15	5 mg/kg rounded to nearest 250mg daily on days 1-7 and 15-21
Level 6	20 mg days 1, 8, 15 and 22	7.5 mg/kg day 1 and 15	10 mg/kg rounded to nearest 250mg daily on days 1-7 and 15-21
Level 7	20 mg days 1, 8, 15 and 22	10 mg/kg day 1 and 15	10 mg/kg rounded to nearest 250mg daily on days 1-7 and 15-21
Level 8	25 mg days 1, 8, 15 and 22	2.5 mg/kg day 1 and 15	5 mg/kg rounded to nearest 250mg daily on days 1-7 and 15-21
Level 9	25 mg days 1, 8, 15 and 22	5 mg/kg day 1 and 15	5 mg/kg rounded to nearest 250mg daily on days 1-7 and 15-21
Level 10	25 mg days 1, 8, 15 and 22	10 mg/kg day 1 and 15	10 mg/kg rounded to nearest 250mg daily on days 1-7 and 15-21

* ***Temsirolimus dosing for the pediatric participants:*** If participants are 40 kg or greater, then the dosing can be the same as for adults. However if <40kg, then at the 5 mg adult dose level, <40kg children be given 0.1

mg/kg temsirolimus; at the 12.5 mg adult dose level, children <40kg get 0.25 mg/kg; and at the 20 mg temsirolimus adult dose level, children <40kg receive 0.4 mg/kg temsirolimus.

TABLE 3: Dose-Escalation Schedule for Bevacizumab/Temsirolimus		
**Dose Level**	**Dose and Schedule (28-day cycle)**	
	**Temsirolimus***	**Bevacizumab**
Level -1	5 mg days 1, 8, 15,and 22	2.5 mg/kg day 1 and 15
Level 0	5 mg days 1, 8, 15 and 22	5 mg/kg day 1 and 15
Level 1	5 mg days 1, 8, 15 and 22	10 mg/kg day 1 and 15
Level 2	12.5 mg days 1, 8, 15 and 22	2.5 mg/kg day 1 and 15
Level 3	12.5 mg days 1, 8, 15 and 22	7.5 mg/kg day 1 and 15
Level 4	12.5 mg days 1, 8, 15 and 22	10 mg/kg day 1 and 15
Level 5	20 mg days 1, 8, 15 and 22	2.5 mg/kg day 1 and 15
Level 6	20 mg days 1, 8, 15 and 22	7.5 mg/kg day 1 and 15
Level 7	20 mg days 1, 8, 15 and 22	10 mg/kg day 1 and 15
Level 8	25 mg days 1, 8, 15 and 22	2.5 mg/kg day 1 and 15
Level 9	25 mg days 1, 8, 15 and 22	5 mg/kg day 1 and 15
Level 10	25 mg days 1, 8, 15 and 22	10 mg/kg day 1 and 15

* ***Temsirolimus dosing for the pediatric participants:*** If participants are 40 kg or greater, then the dosing can be the same as for adults. However if <40kg, then at the 5 mg adult dose level, <40kg children be given 0.1 mg/kg temsirolimus; at the 12.5 mg adult dose level, children <40kg get 0.25 mg/kg; and at the 20 mg temsirolimus adult dose level, children <40kg receive 0.4 mg/kg temsirolimus.

TABLE 4: Regimen Description					
**Agent**	**Premedications; Precautions**	**Dose**	**Route**	**Schedule*****	**Cycle length**
Bevacizumab		** in 100cc NS	Initial dose is infused over 90 minutes. Infusion may be shortened to 60 minutes if the initial infusion is well tolerated. The third and subsequent infusions may be shortened to 30 minutes if the 60 minute infusion is well tolerated.	Day 1 and 15	28 days
Cetuximab	Patients should receive prophylactic intravenous diphenhydramine 25-50mg (or similar antihistamine) approximately 30 minutes before the start of each dose of cetuximab.	**	Loading: IV over 120 min; Maintenance: IV over 60 min; rate not to exceed 5 mL/min	Days 1,8, 15 and 22	28 days
Temsirolimus	CYP3A4 inhibitors. CYP3A4 inducers. Patients should receive prophylactic intravenous diphenhydramine 25-50mg (or similar antihistamine) approximately 30 minutes before the start of each dose of temsirolimus.	** in 250cc of NS	The dose is infused over a 30-60 minute period once a week.	Days 1,8, 15 and 22	28 days
Valproic Acid		**	Oral	Daily on days 1-7 and 15-21	28 days

** Doses as appropriate for assigned dose level.

*** Testing and drug administration will take place as per protocol unless patient/logistical/medical reasons intervene.

7.0 Pretreatment Evaluation

7.1 Complete history and physical examination, including documentation of all measurable disease as well as signs, symptoms, concurrent medications, and performance status.

7.2 Laboratory studies: CBC with differential, sodium, potassium, chloride, bicarbonate, BUN, creatinine, glucose, calcium, magnesium, albumin, alkaline phosphatase, total bilirubin, SGOT[AST], SGPT[ALT], PT/PTT, fasting serum total cholesterol, fasting triglyceride level, serum amylase and lipase (Valproic Acid arm only), urinalysis, serum pregnancy test (women of childbearing potential).

7.3 12-lead EKG within 28 days prior to starting treatment.

7.4 Radiologic evaluation of measurable disease and pertinent tumor markers within 4 weeks before starting treatment. If the patient does not have radiologically measurable disease but has cutaneously measurable disease, this must be documented at the pretreatment evaluation physical examination.

7.5 Optional peripheral blood laboratory studies, including but not limited to VEGF level, soluble VEGFR-2, and VCAM-1 level.

7.6 Optional tumor biopsies for evaluation of angiogenesis-related markers and tumor markers, including but not limited to VEGF, VEGFR-2, phospho-VEGFR-2, HER-2/neu, EGFR, caspase-3, microvessel density, CD 31, p53, Ki67, and hypoxia-inducible factor.

7.7 Optional dynamic contrast-enhanced magnetic resonance imaging (DCE-MRI).

7.8 Patient must sign IRB-approved informed consent prior to any study-specific procedures unless such procedures are part of the standard of care.

8.0 Evaluation During Study

8.1 Physical examination (including vital signs, weight, performance status): weekly physical during cycle 1, then each cycle prior to starting the next cycle.

8.2 Labs weekly during cycle 1, then every week 1 of each cycle: CBC with differential, sodium, potassium, chloride, bicarbonate, BUN, creatinine, glucose, calcium, magnesium, albumin, alkaline phosphatase, total bilirubin, SGOT[AST], SGPT[ALT], fasting total cholesterol, fasting triglyceride level, serum amylase and lipase (Valproic Acid arm only). Urinalysis during week 1 of each cycle.

8.3 Radiologic evaluations and pertinent tumor markers will be repeated after 2

cycles of treatment. The same radiologic method of assessment and the same technique should be used to characterize each identified and reported lesion at baseline and during follow-up.

8.4 Pharmacologic distribution studies will be done by appropriate methods in selected patients when possible.

8.5 Optional peripheral blood laboratory studies, including but not limited to VEGF level, soluble VEGFR-2, and VCAM-1 level.

8.6 Optional tumor biopsies for evaluation of angiogenesis-related markers and tumor markers, including but not limited to VEGF, VEGFR-2, phospho-VEGFR-2, HER-2/neu, EGFR, caspase-3, microvessel density, CD 31, p53, Ki67, and hypoxia-inducible factor.

8.7 Optional dynamic contrast-enhanced magnetic resonance imaging (DCE-MRI).

9.0 Evaluation of Toxicity

9.1 Toxicities will be described according to the NCI-CTCAE Version 3.0. Please refer to Appendix B for the full description of these criteria.

9.2 A dose limiting toxicity (DLT) is defined as a clinically significant adverse event or abnormal laboratory value assessed as unrelated to disease progression, intercurrent illness, or concomitant medications and occurring during the first cycle on study that meets any of the following criteria:

- Grade 3 or 4 non-hematologic toxicity (except nausea and vomiting responsive to appropriate regimens or alopecia)
- Any Grade 4 hematologic toxicity lasting > 14 days despite supportive measures
- Any Grade 4 nausea or vomiting > 5 days despite maximum anti-nausea regimens
- Any other Grade 3 non-hematologic toxicity including symptoms/signs of vascular leak or cytokine release syndrome
- Any severe or life-threatening complication or abnormality not defined in the NCI-CTCAE that is attributable to the therapy

Notes: correctable electrolyte imbalances and alopecia are not considered DLTs.

10.0 Criteria for Response

10.1 While the primary objective of this study is to evaluate dose-ranging experience and the toxicity observed, an attempt to evaluate efficacy will require the following criteria for response. Patients with lymphoma will be measured per the WHO criteria, and all others will be evaluated with the RECIST 1.0 criteria.

11.0 Criteria for Removal from the Study

11.1 Progression of disease per WHO or RECIST criteria as described previously. (Exception: If the patient is deriving clinical benefit from the treatment, then the patient may continue on study at the discretion of the PI).

11.2 The development of unacceptable toxicity.

11.3 Physician recommendation for patient removal from study.

11.4 Patient elects to discontinue further treatment on the study medications.

12.0 Reporting Requirements

12.1 An adverse drug reaction on a Phase I study is a previously unknown or a life-threatening reaction (Grade IV) which may be due to drug administration, or any death on study. The investigator is responsible for the prompt reporting of an Adverse Drug Reaction (ADR) to the Office of Protocol Research (792-2933, Box 1437). This office will in turn notify the Institutional Review Board (IRB).

12.2 Known grade I and II toxicities and all clinically insignificant toxicities will not be tabulated for FDA approved drugs. The study uses FDA approved agents with known toxicity profiles. Therefore, Grade 1 and 2 toxicities (related or unrelated) will not be collected or documented as these are not considered clinically significant in this patient population and/or they are expected for these study agents. Grade 3 and 4 toxicities that are felt to be treatment related and unexpected (per package insert) will be documented. Unless otherwise documented in the electronic medical record as clinically significant and study drug related, all lab abnormalities will be assumed to be related to the patient’s other co-morbid conditions, prior therapies, other concomitant therapies/medications, or underlying cancer. Serious Adverse Events will be reported per standard IRB reporting requirements. Serious unexpected problems will be reported per standard IRB reporting requirements.

13.0 Statistical Considerations

13.1 The primary objective of this study is to assess safety and tolerability as well as to define the maximum tolerated dose of combination treatment with bevacizumab and temsirolimus and/or cetuximab or valproic acid in patients with advanced cancer. A MTD is defined as the dose level below the dose at which 2 of 6 patients experience drug-related dose limiting toxicity (DLT) in the first cycle. Secondary objectives include a preliminary assessment of anti-tumor efficacy of each combination (objective response by RECIST and WHO criteria) and a preliminary assessment of correlation of surrogate anti-angiogenesis markers with anti-tumor activity.

13.2 A dose limiting toxicity (DLT) is defined as a clinically significant adverse event or abnormal laboratory value assessed as unrelated to disease progression, intercurrent illness, or concomitant medications and occurring during the first cycle on study that meets any of the following criteria:

- Grade 3 or 4 non-hematologic toxicity (except nausea and vomiting responsive to appropriate regimens or alopecia)
- Any Grade 4 hematologic toxicity lasting > 14 days despite supportive measures
- Any Grade 4 nausea or vomiting > 5 days despite maximum anti-nausea regimens
- Any other Grade 3 non-hematologic toxicity including symptoms/signs of vascular leak or cytokine release syndrome
- Any severe or life-threatening complication or abnormality not defined in the NCI-CTCAE that is attributable to the therapy

Note: correctable electrolyte imbalances and alopecia are not considered DLTs.

13.3 This protocol will utilize a Phase I escalation design with three to four patients per cohort.

13.4 There will be no intra-patient dose escalation, and no patients will be enrolled in the next dose level until three patients enrolled at the previous dose level have completed at least four weeks of therapy. If a DLT is observed in one of the three patients after one cycle, then dose escalation will not proceed until six patients in the cohort have been assessed for toxicity after one cycle.

13.5 Up to an additional 10 patients with biopsiable disease may be entered at the MTD dose levels after they have been determined for the purpose of exploratory analysis with additional optional correlative studies, including but not limited to dynamic

contrast-enhanced magnetic resonance imaging (DCE-MRI), tumor biopsy, and peripheral blood markers. During expansion, at any time > 33% of patients have a DLT, the expansion cohort will be terminated. If the dose expansion is terminated due to >33% of patients having a DLT, then an additional 10 patients may be entered at the next lowest dose level, for the purpose of exploratory analysis with additional optional correlative studies, including but not limited to dynamic contrast-enhanced magnetic resonance imaging (DCE-MRI), tumor biopsy, and serum/plasma cytokines. For these additional 10 patients, we will monitor toxicity using the same criteria used in the initial expansion cohort.

13.6 Among the patients who undergo DCE-MRI, the tissue microvascular parameters (including K^{trans}, v_e, k_{ep}) will be measured on a continuous scale at the three specified time points. For each of these parameters, an exploratory analysis of change from baseline will be conducted which will include mean, median, standard deviation, and 95% confidence limits.

13.7 We estimate that the number of patients required to find the MTD of this drug combination and to obtain adequate correlative studies is approximately 50-60 patients per arm. The number of patients that **may** be enrolled on the trial, given 11 total dose levels forBevacizumab/Temsirolimus and Cetuximab arm and 10 total dose levels each for Bevacizumab/Temsirolimus and Valproic Acid arm and Bevacizumab/Temsirolimus arm, is 186 (if six patients are enrolled at each dose level). Also, predicting that we will discover 3 MTDs (one for each arm) and allowing for expansion of 10 additional patients at those MTDs, we expect an absolute **maximum of 216 patients for the trial**. In order to enroll 216 evaluable patients, we may need to screen up to 300 patients since we expect a percentage of these patients to be screen failures or withdrawals**.** The estimated accrual rate is 1-5 patients per arm per month.

14.0 Dose Delays and Modifications

14.1 If a patient experiences a toxicity which is known to be related to one drug in the regimen, then that drug may be de-escalated to the prior dose level after the patient recovers to </= Grade 1 toxicity.

14.2 If a patient experiences a toxicity for which it is unclear which drug is the cause of

the toxicity, then the drug which was dose escalated at the current dose level may be de-escalated to the prior dose level after the patient recovers to </= Grade 1 toxicity.

14.3 If a patient experiences a toxicity at the first dose level, and if the toxicity is known to be related to one drug in the regimen, then a dose reduction of 50% of that drug is permitted after the patient recovers to </= Grade 1 toxicity.

14.4 If a patient experiences a toxicity at the first dose level, and if it is unclear which drug is the cause of the toxicity, then a dose reduction of 50% of all drugs in the regimen is permitted after the patient recovers to </= Grade 1 toxicity.

14.5 If grade III toxicity occurs (DLT), dose reduction by 50% is allowed after patient recovers. The drug that will be reduced is the one that the physician feels has most likely caused the toxicity. If the drug that caused the toxicity is not known, the patient will be dose reduced to the previous dose level.

15.0 **Correlative Studies**

15.1 Correlative studies will be mandatory for those patients choosing to enroll in the expansion cohorts of up to 10 patients at the MTDs pending funding.

15.2 Dynamic Contrast-Enhanced Magnetic Resonance Imaging (DCE-MRI)

15.2.1 Dynamic contrast-enhanced magnetic resonance Imaging (DCE-MRI) will be performed at the following time points: 1. Baseline (within one week before day 1 treatment), 2. Acute phase (48 hours +/- 6 hours after first infusion of bevacizumab and temsirolimus); 3. Chronic phase (at end of cycle 1 on day 20). DCE-MRI will be performed at high temporal resolution and will obtain sequential magnetic resonance images before, during, and following the injection of gadolinium (Gd)-chelated contrast medium. Following the acquisition of localizer images, T1 mapping data will be obtained using a multiple flip angle fast spoiled gradient echo sequence in a plane that includes the target lesion(s) as well as a reference vessel. Following the T1 mapping acquisition, the DCE-MRI scans will be obtained using the same pulse sequence and from the same acquisition volume before, during, and following bolus administration of 0.1 mmol/kg gadopentetate dimeglumine at 3 ml/s followed by a 20 ml saline flush also given at 3 ml/s. The total DCE-MRI acquisition will last about 8 minutes. All DCE-MRI data will be analyzed

using a two-compartment model to yield the pharmacokinetic parameters described in Section 2.4 using custom software developed in the IDL and Matlab environments. (52)

15.3 **Pharmacokinetics and Pharmacodynamics:** Optional drug pharmacokinetics may be performed in order to determine the relationship between the pharmacodynamic effects measured by DCE-MRI and the pharmacokinetics of temsirolimus. This approach may contribute to assessing the utility of DCE-MRI as a biomarker of angiogenic activity.

15.3.1 Blood samples will be obtained at the following timepoints during Cycle 1:

1) 10 (+/- 5) min prior to temsirolimus dose
2) 1 hr post temsirolimus dose (+/- 10 min)
3) 4 hr post temsirolimus dose (+/- 10 min)
4) 8 hr post temsirolimus dose (+/- 10 min)
5) 24 - 36 hr post temsirolimus dose (DAY 2)
6) 120 - 168 hr post-temsirolimus dose (DAY 6 - DAY 8); If drawn on DAY 8, must be prior to temsirolimus dose.
7) DAY 15 prior to temsirolimus dose
8) DAY 29 prior to temsirolimus dose

15.3.2 Samples will be analyzed for pharmacokinetics of temsirolimus. Samples will also be analyzed for pharmacodynamics of temsirolimus. Analysis of the pharmacodynamic and pharmacokinetic data will be performed at the Pharmaceutical Development Center at M.D. Anderson Cancer Center using standard methodologies and previously published techniques. Please note that pharmacokinetic data cannot be obtained on bevacizumab as the testing methodologies for this drug are proprietary (Genentech). Pharmacokinetic data cannot be obtained on cetuximab as the testing methodologies for this drug are proprietary (Bristol Myers Squibb).

15.4 **Peripheral Blood Markers of Angiogenesis**

15.4.1 A variety of peripheral blood markers of angiogenesis will be examined at the following timepoints:

<u>For patients doing DCE-MRI:</u>

1) Baseline (within one week prior to Day 1 of Cycle 1)
2) 48 hours +/- 6 hours after dose on Day 1, Cycle 1

3) Day 28-29 of Cycle 1

For patients NOT doing DCE-MRI:

1) Baseline (within two weeks prior to Day 1 of Cycle 1)
2) Day 6-7, Cycle 1
3) Day 28-29 of Cycle 1

15.4.2 Examples include VEGF level, VCAM-1 level, and soluble VEGFR-2. Circulating cytokines will be measured by commercially available ELISAs. Other peripheral blood correlates will be performed in our M.D. Anderson Cancer Center laboratory as per previously published techniques (47).

15.5 **Tumor Biopsies**

15.5.1 Tumor biopsies may be evaluated for a variety of markers at baseline (within 2 weeks prior to infusion) and at approximately the end of cycle 1. Examples include VEGF, VEGFR-2, phospho-VEGFR-2, microvessel density, CD 31, hypoxia-inducible factor-1 (HIF-1), HER2/neu, EGFR, caspase-3, p53, and Ki67. Studies will be performed by immunohistochemistry and standard techniques in our M.D. Anderson Cancer Center laboratory, as previously published. (48)

15.6 **Histone Acetylation in Peripheral Blood Mononuclear cells (PBMC) (optional):**

15.6.1 Optional histone-acetylation assays may be performed in Dr Guillermo Garcia-Manero laboratory at M. D. Anderson Cancer Center. A western Blot for the H3/H4 histone acetylation will be performed on day 1 (before treatment) and day 15 of each cycle. For each day of correlative studies, a blood sample will be withdrawn from the patient and two green-tops tubes will be recovered and sent to Dr Garcia-Manero's laboratory in T6.3821. Please call at 2-7828.

16.0 Study Calendar**

Assessment Tool	Baseline	Cycle 1				Cycle 2 and Subsequent Cycles			
		Week				Week			
		1	2	3	4	1	2	3	4
Temsirolimus*		X	X	X	X	X	X	X	X
Bevacizumab*		X		X		X		X	
Cetuximab OR Valproic Acid*		X***	X	X***	X	X	X	X	X
History/Physical	X	X	X	X	X	X			
CBC with diff	X	X	X	X	X	X			
Basic metabolic panel, Calcium, Magnesium	X	X	X	X	X	X			
Albumin, Liver function tests	X	X	X	X	X	X			
Serum Amylase and Lipase	X	X	X	X	X	X			
Serum Valproic Acid Level		X	X	X	X	X			
Urinalysis	X	X				X			
PT/PTT	X								
Total Cholesterol, Triglyceride	X	X	X	X	X	X			
Serum Pregnancy Test (in women with childbearing potential)	X								
12-lead EKG	X								
Radiologic Reevaluation	X								X
Peripheral Blood Correlative Studies****	X	X			X				
DCE-MRI****	X	X			X				
Pharmacokinetics and Pharmacodynamics****	X	X	X		X				
Tumor Biopsies****	X				X				

* Doses as appropriate for assigned dose level.

**Testing and drug administration will take place as per protocol unless patient/logistical/medical reasons intervene.
*** Valproic acid will be taken daily on days 1-7 and 15-21

**** Optional correlative studies to be done at the expansion of up to 10 patients at the MTD identified for each arm of the study

17.0 References

1. Adjei AA, Hidalgo M: Intracellular signal transduction pathway proteins as targets for cancer therapy. J Clin Oncol 23:5386-403, 2005
2. Baselga J, Arteaga CL: Critical update and emerging trends in epidermal growth factor receptor targeting in cancer. J Clin Oncol 23:2445-59, 2005
3. Blagosklonny MV: Hypoxia-inducible factor: Achilles' heel of antiangiogenic cancer therapy (review). Int J Oncol 19:257-62, 2001
4. Blagosklonny MV: Antiangiogenic therapy and tumor progression. Cancer Cell 5:13-7, 2004
5. Choi KS, Bae MK, Jeong JW, et al: Hypoxia-induced angiogenesis during carcinogenesis. J Biochem Mol Biol 36:120-7, 2003
6. Ferrara N: VEGF as a therapeutic target in cancer. Oncology 69 Suppl 3:11-6, 2005
7. Hicklin DJ, Ellis LM: Role of the vascular endothelial growth factor pathway in tumor growth and angiogenesis. J Clin Oncol 23:1011-27, 2005
8. Lee JW, Bae SH, Jeong JW, et al: Hypoxia-inducible factor (HIF-1)alpha: its protein stability and biological functions. Exp Mol Med 36:1-12, 2004
9. Tsuzuki Y, Fukumura D, Oosthuyse B, et al: Vascular endothelial growth factor (VEGF) modulation by targeting hypoxia-inducible factor-1alpha--> hypoxia response element--> VEGF cascade differentially regulates vascular response and growth rate in tumors. Cancer Res 60:6248-52, 2000
10. Yu JL, Rak JW, Coomber BL, et al: Effect of p53 status on tumor response to antiangiogenic therapy. Science 295:1526-8, 2002
11. Zhong H, De Marzo AM, Laughner E, et al: Overexpression of hypoxia-inducible factor 1alpha in common human cancers and their metastases. Cancer Res 59:5830-5, 1999
12. Powis G, Kirkpatrick L: Hypoxia inducible factor-1alpha as a cancer drug target. Mol Cancer Ther 3:647-54, 2004
13. Escuin D, Simons JW, Giannakakou P: Exploitation of the HIF axis for cancer therapy. Cancer Biol Ther 3:608-11, 2004
14. Welsh SJ, Koh MY, Powis G: The hypoxic inducible stress response as a target for cancer drug discovery. Semin Oncol 33:486-97, 2006
15. Welsh SJ, Powis G: Hypoxia inducible factor as a cancer drug target. Curr Cancer Drug Targets 3:391-405, 2003
16. Kim MS, Kwon HJ, Lee YM, et al: Histone deacetylases induce angiogenesis by negative regulation of tumor suppressor genes. Nat Med 7:437-43, 2001
17. Qian DZ, Wang X, Kachhap SK, et al: The histone deacetylase inhibitor NVP-LAQ824 inhibits angiogenesis and has a greater antitumor effect in combination with the vascular endothelial growth factor receptor tyrosine kinase inhibitor PTK787/ZK222584. Cancer Res 64:6626-34, 2004
18. Bianco R, Garofalo S, Rosa R, et al: Inhibition of mTOR pathway by everolimus cooperates with EGFR inhibitors in human tumours sensitive and resistant to anti-EGFR drugs. Br J Cancer 98:923-30, 2008
19. Beaudry P, Force J, Naumov GN, et al: Differential effects of vascular endothelial growth factor receptor-2 inhibitor ZD6474 on circulating endothelial progenitors and mature circulating endothelial cells: implications for use as a surrogate marker of antiangiogenic activity. Clin Cancer Res 11:3514-22, 2005

20. Folkman J: Tumor angiogenesis: therapeutic implications. N Engl J Med 285:1182-6, 1971

21. Burger RA, Sill MW, Monk BJ, et al: Phase II trial of bevacizumab in persistent or recurrent epithelial ovarian cancer or primary peritoneal cancer: a Gynecologic Oncology Group Study. J Clin Oncol 25:5165-71, 2007

22. Hurwitz H, Fehrenbacher L, Novotny W, et al: Bevacizumab plus irinotecan, fluorouracil, and leucovorin for metastatic colorectal cancer. N Engl J Med 350:2335-42, 2004

23. Johnson DH, Fehrenbacher L, Novotny WF, et al: Randomized phase II trial comparing bevacizumab plus carboplatin and paclitaxel with carboplatin and paclitaxel alone in previously untreated locally advanced or metastatic non-small-cell lung cancer. J Clin Oncol 22:2184-91, 2004

24. Miller KD, Chap LI, Holmes FA, et al: Randomized phase III trial of capecitabine compared with bevacizumab plus capecitabine in patients with previously treated metastatic breast cancer. J Clin Oncol 23:792-9, 2005

25. Yang JC, Haworth L, Sherry RM, et al: A randomized trial of bevacizumab, an anti-vascular endothelial growth factor antibody, for metastatic renal cancer. N Engl J Med 349:427-34, 2003

26. Davis DW, McConkey DJ, Abbruzzese JL, et al: Surrogate markers in antiangiogenesis clinical trials. Br J Cancer 89:8-14, 2003

27. Hylton N: Dynamic contrast-enhanced magnetic resonance imaging as an imaging biomarker. J Clin Oncol 24:3293-8, 2006

28. Miller JC, Pien HH, Sahani D, et al: Imaging angiogenesis: applications and potential for drug development. J Natl Cancer Inst 97:172-87, 2005

29. Padhani AR, Leach MO: Antivascular cancer treatments: functional assessments by dynamic contrast-enhanced magnetic resonance imaging. Abdom Imaging 30:324-41, 2005

30. Rehman S, Jayson GC: Molecular imaging of antiangiogenic agents. Oncologist 10:92-103, 2005

31. Del Bufalo D, Ciuffreda L, Trisciuoglio D, et al: Antiangiogenic potential of the Mammalian target of rapamycin inhibitor temsirolimus. Cancer Res 66:5549-54, 2006

32. Fidler IJ, Ellis LM: The implications of angiogenesis for the biology and therapy of cancer metastasis. Cell 79:185-8, 1994

33. Carmeliet P: VEGF as a key mediator of angiogenesis in cancer. Oncology 69 Suppl 3:4-10, 2005

34. Ferrara N, Hillan KJ, Novotny W: Bevacizumab (Avastin), a humanized anti-VEGF monoclonal antibody for cancer therapy. Biochem Biophys Res Commun 333:328-35, 2005

35. Glickman MH, Ciechanover A: The ubiquitin-proteasome proteolytic pathway: destruction for the sake of construction. Physiol Rev 82:373-428, 2002

36. Ferrara N, Hillan KJ, Gerber HP, et al: Discovery and development of bevacizumab, an anti-VEGF antibody for treating cancer. Nat Rev Drug Discov 3:391-400, 2004

37. Duda DG, Cohen KS, di Tomaso E, et al: Differential CD146 expression on circulating versus tissue endothelial cells in rectal cancer patients: implications for circulating endothelial and progenitor cells as biomarkers for antiangiogenic therapy. J Clin Oncol 24:1449-53, 2006

38. Jordan BF, Runquist M, Raghunand N, et al: Dynamic contrast-enhanced and diffusion MRI show rapid and dramatic changes in tumor microenvironment in response to inhibition of HIF-1alpha using PX-478. Neoplasia 7:475-85, 2005

39. Kane RC, Farrell AT, Sridhara R, et al: United States Food and Drug Administration approval summary: bortezomib for the treatment of progressive multiple myeloma after one prior therapy. Clin Cancer Res 12:2955-60, 2006
40. Adams J: The proteasome: structure, function, and role in the cell. Cancer Treat Rev 29 Suppl 1:3-9, 2003
41. Dancey JE: Clinical development of mammalian target of rapamycin inhibitors. Hematol Oncol Clin North Am 16:1101-14, 2002
42. Bhalla KN: Epigenetic and chromatin modifiers as targeted therapy of hematologic malignancies. J Clin Oncol 23:3971-93, 2005
43. Agalioti T, Chen G, Thanos D: Deciphering the transcriptional histone acetylation code for a human gene. Cell 111:381-92, 2002
44. de la Cruz X, Lois S, Sanchez-Molina S, et al: Do protein motifs read the histone code? Bioessays 27:164-75, 2005
45. Kouraklis G, Theocharis S: Histone deacetylase inhibitors and anticancer therapy. Curr Med Chem Anticancer Agents 2:477-84, 2002
46. Yang H, Hoshino K, Sanchez-Gonzalez B, et al: Antileukemia activity of the combination of 5-aza-2'-deoxycytidine with valproic acid. Leuk Res 29:739-48, 2005
47. Phiel CJ, Zhang F, Huang EY, et al: Histone deacetylase is a direct target of valproic acid, a potent anticonvulsant, mood stabilizer, and teratogen. J Biol Chem 276:36734-41, 2001
48. Sekulic A, Hudson CC, Homme JL, et al: A direct linkage between the phosphoinositide 3-kinase-AKT signaling pathway and the mammalian target of rapamycin in mitogen-stimulated and transformed cells. Cancer Res 60:3504-13, 2000
49. Zhang X, Shu L, Hosoi H, et al: Predominant nuclear localization of mammalian target of rapamycin in normal and malignant cells in culture. J Biol Chem 277:28127-34, 2002
50. Barbet NC, Schneider U, Helliwell SB, et al: TOR controls translation initiation and early G1 progression in yeast. Mol Biol Cell 7:25-42, 1996
51. Schmelzle T, Hall MN: TOR, a central controller of cell growth. Cell 103:253-62, 2000
52. Geoerger B, Kerr K, Tang CB, et al: Antitumor activity of the rapamycin analog CCI-779 in human primitive neuroectodermal tumor/medulloblastoma models as single agent and in combination chemotherapy. Cancer Res 61:1527-32, 2001
53. Brown EJ, Albers MW, Shin TB, et al: A mammalian protein targeted by G1-arresting rapamycin-receptor complex. Nature 369:756-8, 1994
54. Brown EJ, Schreiber SL: A signaling pathway to translational control. Cell 86:517-20, 1996
55. Terada N, Patel HR, Takase K, et al: Rapamycin selectively inhibits translation of mRNAs encoding elongation factors and ribosomal proteins. Proc Natl Acad Sci U S A 91:11477-81, 1994
56. Dudkin L, Dilling MB, Cheshire PJ, et al: Biochemical correlates of mTOR inhibition by the rapamycin ester CCI-779 and tumor growth inhibition. Clin Cancer Res 7:1758-64, 2001
57. Hudes G, Carducci M, Tomczak P, et al: Temsirolimus, interferon alfa, or both for advanced renal-cell carcinoma. N Engl J Med 356:2271-81, 2007
58. Raymond E, Alexandre J, Faivre S, et al: Safety and pharmacokinetics of escalated doses of weekly intravenous infusion of CCI-779, a novel mTOR inhibitor, in patients with cancer. J Clin Oncol 22:2336-47, 2004

59. Atkins MB, Hidalgo M, Stadler WM, et al: Randomized phase II study of multiple dose levels of CCI-779, a novel mammalian target of rapamycin kinase inhibitor, in patients with advanced refractory renal cell carcinoma. J Clin Oncol 22:909-18, 2004

60. Chang SM, Kuhn J, Wen P, et al: Phase I/pharmacokinetic study of CCI-779 in patients with recurrent malignant glioma on enzyme-inducing antiepileptic drugs. Invest New Drugs 22:427-35, 2004

61. Margolin K, Longmate J, Baratta T, et al: CCI-779 in metastatic melanoma: a phase II trial of the California Cancer Consortium. Cancer 104:1045-8, 2005
